# Cancer-Associated Fibroblast Heterogeneity, Activation and Function: Implications for Prostate Cancer

**DOI:** 10.3390/biom13010067

**Published:** 2022-12-29

**Authors:** Jasmine S. Owen, Aled Clayton, Helen B. Pearson

**Affiliations:** 1 The European Cancer Stem Cell Research Institute, School of Biosciences, Cardiff University, Cardiff CF24 4HQ, UK; 2 Tissue Microenvironment Group, Division of Cancer & Genetics, School of Medicine, Cardiff University, Cardiff CF14 4XN, UK

**Keywords:** fibroblast, prostate cancer, cancer-associated fibroblasts, tumor microenvironment

## Abstract

The continuous remodeling of the tumor microenvironment (TME) during prostate tumorigenesis is emerging as a critical event that facilitates cancer growth, progression and drug-resistance. Recent advances have identified extensive communication networks that enable tumor–stroma cross-talk, and emphasized the functional importance of diverse, heterogeneous stromal fibroblast populations during malignant growth. Cancer-associated fibroblasts (CAFs) are a vital component of the TME, which mediate key oncogenic events including angiogenesis, immunosuppression, metastatic progression and therapeutic resistance, thus presenting an attractive therapeutic target. Nevertheless, how fibroblast heterogeneity, recruitment, cell-of-origin and differential functions contribute to prostate cancer remains to be fully delineated. Developing our molecular understanding of these processes is fundamental to developing new therapies and biomarkers that can ultimately improve clinical outcomes. In this review, we explore the current challenges surrounding fibroblast identification, discuss new mechanistic insights into fibroblast functions during normal prostate tissue homeostasis and tumorigenesis, and illustrate the diverse nature of fibroblast recruitment and CAF generation. We also highlight the promise of CAF-targeted therapies for the treatment of prostate cancer.

## 1. Introduction

Prostate cancer is a leading cause of death in men globally [1], reflecting our poor molecular understanding of this malignancy and the limited efficacy of standard treatment regimens against advanced disease [2]. Prostate cancer is generally thought of as a hormone-driven disease, dependent on androgen-mediated androgen receptor (AR) signaling for growth [3]. This has led to the use of androgen-deprivation therapy (ADT)/AR-signaling inhibitors (ARSi) as a standard treatment regimen for prostate cancer, which is often administered in combination with chemotherapy, radiotherapy and/or immunotherapy [3]. A large number of patients initially respond to androgen/AR-directed therapies, however over time patients inevitably relapse as tumor cells transform and become resistant to treatment, termed castrate-resistant prostate cancer (CRPC) [4]. Advanced CRPC is currently incurable, highlighting the need for more effective therapies and novel biomarkers. While the molecular mechanisms that facilitate CRPC transition and progression remain to be fully defined, increasing evidence has indicated that the surrounding TME plays a central role in supporting tumor growth and dissemination [5,6].

The TME consists of a plethora of different components, including stromal fibroblasts and their secretome components, extracellular matrix (ECM), immune and inflammatory cells and endothelial cells. Bidirectional communication between the tumor and the surrounding stroma within the prostate is integral for cancer progression [7,8]. This cross-talk is largely achieved through the release of a cocktail of signaling molecules, including growth factors, cytokines, chemokines and extracellular vesicles (EVs), however the extent of this complex communication network within prostate cancer remains unclear [7,8]. Moreover, increasing evidence in the literature suggests that tumor–stroma interactions can continually adapt, as the TME and the tumor cells co-evolve to overcome physiological and environmental stress [9].

Expansion of the stromal compartment has been observed during all stages of prostate cancer progression and is particularly profound in advanced disease. Nonetheless, the status of stromal reactivity is currently not considered by histopathologists when assigning tissue-grading scores [10]. CAFs constitute a major component of the TME and extensive research efforts have revealed that CAFs have the ability to regulate the TME composition and influence the behavior of malignant and immune cells to regulate prostate cancer growth and invasive progression by remodeling the ECM, inducing angiogenesis and secreting multiple signaling molecules [11,12,13,14,15]. Recent research has also begun to highlight the existence of a diverse range of CAF subpopulations with unique functions that can evolve throughout tumorigenesis [11,12,13,14,15]. Here, we review recent findings with respect to the origin, function and identification of fibroblasts and CAFs to better understand their role during normal adult prostate tissue homeostasis and tumorigenesis, and explore the latest advances in targeting CAFs to treat prostate cancer.

### 1.1. Fibroblasts Function to Maintain Normal Adult Prostate Tissue Homeostasis

Fibroblasts are generally derived from the mesoderm during embryogenesis and are critical for mammalian development and normal adult tissue homeostasis [15]. To maintain tissue homeostasis, fibroblasts primarily function to synthesize and turnover ECM components via the balanced secretion of modifying enzymes, such as matrix metalloproteinases (MMPs) and tissue inhibitors of MMPs (TIMPs) [16]. The nuanced remodeling activity of fibroblasts results in an array of distinct, tissue-specific ECMs throughout the body, enabling stromal support for specialized cells of different organs [17]. Even though fibroblasts play a common cross-organ function in ECM remodeling, remarkably single-cell RNA sequencing (scRNAseq) analysis of fibroblasts in normal adult mouse heart, skeletal muscle, intestine and bladder has shown that there is a less than 20% overlap between the fibroblast transcriptome in these tissues, indicating a vast degree of fibroblast inter-tissue heterogeneity exists in addition to tissue-specific specialties [18].

Although fibroblasts are largely quiescent, they can be activated to restore normal tissue homeostasis in response to extrinsic cues (such as growth factors, cytokines, chemokines, mechanical and other forms of physical stress or tissue damage) [13,19,20]. In response to tissue damage, activated fibroblasts can rapidly proliferate to orchestrate the remodeling of the ECM and a healing response [14,21]. Recently, fibroblasts have also been shown to play roles in immune surveillance and inflammation, blood vessel formation, maintenance of the stem cell niche and cancer progression [11,14]. In a non-malignant adult prostate, diverse subpopulations of resting and activated fibroblasts maintain tissue architecture and function by coordinating distinct events that remain to be fully defined [11,22,23,24,25,26].

#### 1.1.1. Fibroblasts Cross-Talk with Prostate Epithelial Cells

While the underlying mechanisms whereby fibroblasts maintain normal tissue homeostasis in the adult prostate remain to be elucidated, previous work has shown that normal prostate fibroblasts express AR, and previous tissue recombination studies using urogenital sinus mesenchyme and prostate epithelium have revealed that stromal AR is essential for prostate organogenesis [27,28]. Moreover, deletion of *AR* within prostatic smooth muscle cells or fibroblasts in mice has been shown to reduce prostate weight and predispose to prostate hyperplasia, inflammation and fibrosis, as well as influencing prostate epithelial cell proliferation, apoptosis and differentiation [29,30,31]. Together these data indicate that stromal/fibroblastic AR is required for normal prostate tissue homeostasis in vivo.

In addition to AR, prostate fibroblasts may also maintain normal adult prostate tissue homeostasis via Wnt signaling. The Wnt pathway regulates epithelial cell proliferation, survival, migration and stem cell activity during mammalian development, and oncogenic Wnt signaling is a common event in several human malignancies, including prostate cancer [32]. Recently, Wnt signaling has been reported to mediate fate specification of fibroblast progenitors into numerous lineages, including fibroblasts in the skin [33], and tumor growth factor β (TGFβ)-Wnt signaling cross-talk has been shown to regulate myofibroblast activation in skin by reducing the Wnt antagonist Dikkopf-1 (DKK1) [34]. RNAseq analysis of Lin^−^CD24^−^CD49f^−^Sca-1^+^ stromal cells isolated from the proximal region of an adult mouse prostate (a mouse prostate stem cell niche) are reported to display upregulation of multiple Wnt ligands and Wnt receptors/co-receptors relative to the distal region and display high Wnt/β-catenin activity [35]. Proximal stromal cell-derived Wnt5a (a non-canonical Wnt ligand) was also found to suppress prostate epithelial stem/progenitor cell proliferation via TGFβ paracrine signaling [35]. Importantly, systemic expression of stabilized β-catenin in stromal mouse cells (driven by *Col1a2Cre-ERT2*) caused a reduction in total body weight and prostate weight associated with reduced prostate epithelial cell proliferation, which was phenocopied in prostate tissue reconstitution assays in the renal capsule [35]. In contrast, loss of β-catenin in stromal cells increased prostate epithelial cell proliferation in adult mice, indicating that activated Wnt signaling in stromal cells reduces prostate epithelial cell proliferation in this setting.

Despite the limited research exploring PDGF signaling and normal prostate fibroblasts, platelet-derived growth factor receptor alpha/beta (PDGFRα/β) have been identified in several fibroblasts and are considered to be commonly expressed on the surface of normal prostate fibroblasts [11,24,35,36]. Given that PDGF signaling in fibroblasts can be activated by an autocrine process or via secretion of the PDGF ligand by epithelial cells [37,38], it is possible that PDGF pathway activity may play a role in maintaining normal adult prostate tissue homeostasis. Nevertheless, further work is needed to investigate this possibility. In support of this concept, functional genetic studies have reported that stromal PDGFRα is essential for embryonic development and tissue homeostasis in multiple tissues including the heart and lung [39,40,41,42].

#### 1.1.2. The Role of Fibroblasts during Ageing

While the relationship between fibroblasts and age-related physiological and cellular changes has not been well characterized in the prostate, aged fibroblasts have been shown to acquire a phenotype which is indicative of senescence [43]. Moreover, aged fibroblasts are reported to exhibit growth arrest and are more resistant to apoptotic cues [43]. For instance, in contrast to young fibroblasts senescent human fibroblasts are reported to accumulate ceramides that regulate apoptosis, and are more resistant to ceramide-induced apoptosis in vitro [44]. Senescent human fibroblasts have also been reported to resist apoptosis caused by growth factor deprivation and reactive oxygen species (ROS) accumulation [45,46]. Furthermore, aged fibroblasts can induce a state of chronic tissue inflammation via their senescence-associated secretome that contains MMPs, growth factors and ROS, which can subsequently alter the integrity of the elastin network [45,46]. In combination, inflammation and loss of elasticity can increase tissue stiffness and reduce mechanical strength [47], potentially facilitating the creation of a tumor permissive environment.

#### 1.1.3. Normal Fibroblasts Facilitate Wound Repair and Inflammation

Resting fibroblasts can become activated and differentiate into myofibroblasts that are characterized by the co-expression of α-smooth muscle actin (αSMA) positive stress fibers and vimentin in response to a complex range of stress stimuli [13,14]. These include changes in tensile force and secretion of a variety of factors by damaged epithelial cells and infiltrating immune cells, such as damage-associated molecular patterns (DAMPs), growth factors (e.g., TGFβ, PDGFs, and fibroblast growth factors (FGFs)) and cytokines (e.g., interferon gamma (IFNγ), tumor necrosis factor alpha (TNFα) and interleukins (ILs)) [11,19,22,48]. In the resting state, the production of ECM components (e.g., collagen and elastin) by fibroblasts is minimal; however, upon transition into a contractile myofibroblast state there is significant ECM remodeling owing to their elevated and altered production of ECM constituents and ECM remodeling enzyme secretion (e.g., increased collagen/Tenascin C (TNC) production and increased hyaluronan cross-links) [49].

TGFβ signaling is perhaps one of the best characterized mechanisms of myofibroblast activation. Upon exposure to a wound, myofibroblasts can be activated via the upstream activation of the transforming growth factor beta receptors 1/2 (TGFβR1/2) expressed on their cell surface (reviewed in [22]). Activated myofibroblasts can initiate a tissue remodeling cascade through coordination of immune cell activity [50], induction of angiogenesis [51], mediation of stem cell behavior [52], and contraction of granulation tissue for wound closure [53]. During wound repair, myofibroblasts can promote inflammation by up-regulating ECM production, which facilitates immune cell recruitment and cytokine release (e.g., TGFβ, IL-1B, C-X-C motif (CXC) and C-C motif (CC) chemokines) [35,36,54,55,56,57]. These signaling molecules can also activate resident immune cells, such as macrophages, attracting them to the site of damage [57]. Accordingly, sustained activation of myofibroblasts has also been linked to chronic inflammation, known as fibrosis [58]. Benign prostate hyperplasia (BPH) is also associated with reactive stroma, which is similar to a wound repair response commonly seen in inflammation-induced fibrosis (reviewed in [59]). BPH is reported to harbor a large population of myofibroblasts that have been implicated in driving the reactive stromal response [59], however further work is needed to determine the functional significance of myofibroblasts during BPH growth.

### 1.2. Identification of Fibroblasts in Normal Adult Prostate Tissue

Substantial research efforts have been undertaken to identify fibroblasts in epithelial tissues during normal adult tissue homeostasis, with a view to improving our molecular understanding of how fibroblasts function to regulate tissue homeostasis and to gain new insights into their origins. This work has led to the identification of a range of fibroblast populations that reside in normal tissues, including the prostate (summarized in Table 1). To date, four normal fibroblast subtypes have been identified using a range of protein and RNA markers; however, many of these markers are also present on other cells resident within the tissue microenvironment, including smooth muscle cells and endothelial cells. Unfortunately, due to the vast heterogeneity of fibroblasts, the extent to which these subpopulations of fibroblast overlap remains to be determined, and new markers, or combinations of markers, that are unique to fibroblasts are needed to avoid confusing other cell types with fibroblasts.

**Table 1:** Overview of normal adult prostate tissue fibroblasts markers, function and cell-of-origin.

Cell Type (Species)	Alternative Nomenclature	Protein and mRNA Expression Markers ^1^	Functions	Cell-of-Origin	Reference ^2^
**Fibroblasts** (Human and Mouse)	Resting fibroblasts or Quiescent adult Fibroblasts	VIM, PDGFR-α, PDPN, αSMA, FSP1, NG2, Col1a1, DCN, *STC1*, *NKTR*, *CAV1*, *CFH*, *Fn*, *PARVA*, *SPARC*, *Col1a2*, *Col5a1*, *Fbln1/2*, *Cd34*, *FGF2*, *FGF7*, *SNAPC2*, *AR*, *S100A16*, *FAP*, *ANXA2P3*, *SPRED2*, *PTGDS*, *RSPO3*	Maintain normal tissue homeostasis ECM deposition	Mesenchymal cells	**[23,24,25,26]** Breast and pancreas: [60,61] Muscular organs: [18] Cervix [62]
**Periacinar** **Fibroblasts** (Human)	Periacinar myofibroblasts	VIM, αSMA, COL1A1, COL3A1, FN, P4HB, COL4, Laminin, NMII	Maintain normal tissue homeostasis ECM deposition	Mesenchymal cells	**[25]** Pancreas: [63]
**Fibrocytes** (Human)	Bone marrow-derived cells, fibroblasts or myofibroblasts	CD45, COL1A1	Collage type-I production Inflammatory secretome production during fibrosis/scarring	Bone-marrow-derived mesenchymal stem cells	Liver: [13,64]
**Myofibroblasts** (Human and Mouse)	Activated fibroblasts or Normal activated fibroblasts	VIM, αSMA, FAP, PDPN, AR, TGFβR1/2, FSP1, COL1A1, COL3A1, CFH, *PARVA*, *SPARC*, *pSMAD2/3*, *FN*, *TNC*	Wound healing	Resident fibroblasts (in response to TGF-β)	**[25,65,66,67]** (reviewed by **[11,22,68]**)
**Smooth muscle cells** (Human)		αSMA, CNN1	Maintain normal tissue homeostasis (AR signaling)	Mesenchymal cells	**[23,25]**
**Fibromuscular stromal cells** (Human)		CD49a, CD49e, CD51/61, CD30, CD29, CD55, CD56, CD59, CD79, CD81, CD90, CD99, CD131	Maintain normal tissue homeostasis	Mesenchymal cells	**[69]**
**Endothelial cells** (Human and Mouse)		VIM, CD31, CD34, CD105, VEGFR, CD200	Forms a layer that lines blood vessels Modulates exchanges (e.g., signal molecules, EVs, gases) between the bloodstream and surrounding tissues.	Mesenchymal cells	**[23]** (reviewed by **[70]**) Pancreas: [61]

^1^ Gray text = expression marker identified but not consistently expressed. ^2^ Bold references = literature specific to prostate tissue. αSMA = alpha smooth muscle actin; ANXA2P3 = annexin A2 pseudogene 3; AR = androgen receptor; ASPN = asporin; CNN1 = Calponin; CAV1 = Calveolin-1; CD26 = Dipeptidyl peptidase; CD29 = Integrin β1; CD30 = TNF receptor superfamily member 8; CD31 = Platelet endothelial cell adhesion molecule; CD34 = CD34 molecule; CD49a/e = integrin subunit alpha 1; CD51/61 = integrin subunit alpha V/beta 3; CD55 = CD55 molecule (cromer blood group); CD56 = Neural cell adhesion molecule 1; CD59 = CD59 molecule blood group; CD63 = CD63 molecule; CD79 = CD79 molecule; CD90 = Thy-1 pr cluster of differentiation 90; CD99 = CD99 molecule Xg blood group; CD105 = Endoglin; COL1A1/2 = collagen type I alpha 1/2 chain; CD131 = Colony stimulating factor 2 receptor subunit beta; DCN = Decorin; CTSK = cathepsin K; ECM = extracellular matrix; EGF = epidermal growth factor; FAP = fibroblast activation protein; FBLN1 = Fibulin 1; FGF2/7/10 = fibroblast growth factor-2/7/10; FN = fibronectin; FSP1 = fibroblast secretory protein 1; HGF = hepatocyte growth factor; IGF1 = insulin growth factor-1; NG2 = Neuron-glial antigen 2; NKTR = Natural killer cell triggering receptor; PARVA = Parvin alpha; PDPN = Podoplanin; PDGFR-α/β = platelet-derived growth factor receptor alpha/beta; pSMAD2/3 = phospho-Mothers against decapentaplegic homolog 2/3; S100A16 = S100 calcium binding protein A16; SPANC2 = Small nuclear RNA activating complex polypeptide 2; SPARC = Secreted protein acidic and cysteine rich; SPRED2 = sprouty-related EVH1 domain containing 2; STC1 = staneocalcin1; TGFβ = Transforming growth factor β; TGFβR1/2 = Transforming growth factor β receptor 1/2; TNC = Tenascin; VEGF = vascular endothelial growth factor; VIM = vimentin; ZEB1 = Zinc Finger E-Box Binding Homeobox 1.

Until recently, there has been very little progress in identifying normal prostate fibroblast markers that are specific, limiting our understanding of their function, localization and distribution. Advances in the field using gene expression profiling, real-time quantitative reverse transcription polymerase chain reaction (QRTPCR) and immunofluorescent staining have revealed a panel of candidate markers, including vimentin, αSMA, fibroblast specific protein 1 (FSP1), AR, small nuclear RNA activating complex polypeptide 2 (SNAPC2), S100 calcium binding protein A16 (S100A16); annexin A2 pseudogene 3 (ANXA2P3), and sprouty-related EVH1 domain containing 2 (SPRED2) [23,24,26] (Table 1). Nevertheless, the true spectrum of fibroblasts in non-malignant adult prostate tissue and during prostate disease remains to be determined. It is therefore paramount that future studies undertake a comprehensive assessment of fibroblast populations in the prostate to take us a step closer to developing species-specific classification criteria for normal fibroblast subtypes, and improve our molecular understanding of how these diverse cells are regulated and their mode of action during prostatitis, BPH and prostate cancer.

## 2. Prostate Cancer-Associated Fibroblast Origin, Activation and Recruitment

CAFs are a group of dynamically heterogenous cells of predominantly mesenchymal origin, with distinct functions orchestrated by their secretome and regulation of cell signaling networks that enable cross-talk between CAFs, other TME resident cells and cancer cells [13,71,72,73]. The establishment of diverse CAF subpopulations is influenced by a multitude of factors, including tumor stage, location, oncogenic cell signaling from cancer cells, the CAF cell-of-origin, the ECM composition and the immune response [13,71,72,73]. Pro-tumorigenic functions include angiogenesis, promotion of epithelial cell proliferation and creation of an immunosuppressive TME [13,71,72,73]. However, it is important to note that normal stromal fibroblasts and CAFs during the early stages of malignancy have also been shown to suppress tumor growth [14], adding further complexity to the role of CAFs during tumorigenesis. Hence paradoxically, while CAF activation can occur during the early stages of oncogenesis, activated CAFs have the potential to exert tumor suppressive functions in this setting.

It is currently speculated that during tumor growth and progression, cancer cells and CAFs can dynamically co-evolve, supporting one another via reciprocal feedback loops involving several molecules (e.g., cytokines, growth factors and extracellular vesicles) to promote tumor growth, cell migration/invasion, ECM remodeling and pro-tumorigenic microenvironment alterations such as angiogenesis and immune evasion [11,14,25,74]. In turn, tumor-derived growth factors and cytokines can stimulate CAF tumor-promoting activities, presenting a direct mechanism whereby CAF activity status, location, recruitment and subtype continually co-evolve as the tumor progresses to support tumor growth [6,75,76,77]. CAF-tumor co-evolution is likely to be dependent on a variety of factors, including the tumor type, rate of tumor progression, and stromal composition [14]. Additional research is needed to better understand the mechanistic basis underpinning this process and will require robust approaches that can accurately identify CAF subtypes.

### 2.1. The Heterogeneous Origin of CAFs in Prostate Cancer

Although the origin of CAFs remains unclear in the literature, several studies have indicated that CAFs display lineage plasticity and originate from a diverse population of cells residing in the TME through differentiation processes that are poorly understood. While the predominant prostate CAF cell-of-origin is generally considered to stem from the expansion and activation of the resident fibroblasts, CAF recruitment from other sources has also been reported in the literature [14,78]. For instance, epithelial cells that have undergone epithelial-to-mesenchymal transition (EMT) have been shown to undergo CAF transition, and mesenchymal stem cells, fibrocytes and circulating bone marrow-derived cells are also reported to home to the prostate and differentiate into CAFs [13,20,79,80,81,82,83,84,85,86]. CAF-like hybrid cell populations may also exist in some malignancies originating from pericytes, vascular mural cells, and endothelial cells [13,87,88]. To develop our understanding of the diverse nature of CAF generation in prostate cancer, we have surveyed the literature to illustrate the broad range of CAF origins.

#### 2.1.1. Resident Fibroblasts within the TME Differentiate into CAFs

During normal, non-malignant circumstances, myofibroblasts that have facilitated wound repair will typically undergo programed cell death, enabling the microenvironment to return to its resting state [53]. Interestingly, histological features of a wound are often mirrored in the TME, thus coining the expression “tumors are wounds that do not heal” [89]. Consequently, during malignant growth myofibroblasts may become activated similarly to the wound healing process, however unlike wound healing they can evade apoptosis via continued exposure to growth factors and cytokines, and continue to proliferate and evolve into a heterogeneous population of CAFs [13,90]. In prostate cancer, myofibroblast survival is predominantly initiated by the release of TGFβ from epithelial cells, which in turn causes myofibroblasts to also secrete TGFβ, potentiating a positive feedback loop [11,48,91]. Metabolic reprogramming is also required for fibroblasts-to-CAF differentiation, which is influenced by additional tumor-derived factors (e.g., ILs and microRNAs) [12]. Together, these events lead to the development of a reactive stroma that is maintained by the ECM remodeling capabilities of the CAFs, and helps to fuel a vicious cycle of tumor growth and CAF activation, proliferation and migration [90].

In prostate cancer, it is generally considered that resident fibroblasts differentiate into the majority of CAFs observed [14,78]. Several groups have shown through transcriptomic and proteomic analysis that the majority of CAFs present in both the human and murine TME express high levels of vimentin, a marker which is also highly expressed in normal fibroblasts [24,36,57,60,61]. However, vimentin expression may also be high in other cells of mesenchymal origin (e.g., mesenchymal stem cells and fibrocytes), and is a surrogate marker for EMT in prostate cancer [92]. Consequently, distinguishing exactly which cell types are vimentin-positive within a tumor remains a current challenge. Furthermore, PDGFRα and FSP1 fibroblast markers are also highly expressed in multiple CAF subpopulations identified in prostate cancer [36,93,94,95] (discussed in Section 3). Similarly, melanoma CAFs also often express high PDGFRα and are predicted to be derived from local normal fibroblasts [96].

#### 2.1.2. Local Mesenchymal Cell Derived CAFs

Mesenchymal cells derived from the embryonic mesoderm have also been shown to differentiate into CAFs. In prostate cancer, a number of paracrine factors have been found to be upregulated in both the stroma and the epithelial cells, and can stimulate growth and expansion of both compartments. These factors include, FGF, PDGFRα/β, TGFβ, epidermal growth factor (EGF) and insulin growth like factor (IGF) family receptors and are implicated in mesenchymal cell functions, suggesting that they are a CAF cell-of-origin [97]. Notably, FGF10 is highly expressed in the stroma of well-differentiated tumors and is essential for normal prostate development, where it is primarily expressed in mesenchymal cells [98,99]. Furthermore, Memarzadeh and colleagues found that enhanced mesenchymal expression of FGF10 causes multifocal prostate-intraepithelial neoplasia or adenocarcinoma in mice attributable to FGF10 paracrine signaling, and this phenotype was rescued by attenuating fibroblast growth factor receptor-1 or -2 (FGFR1 or FGFR2) signaling in prostate epithelial cells using dominant-negative FGFR1/2 constructs [100]. Taken together, these studies indicate the pro-tumorigenic function of elevated levels of mesenchymal FGF10 and suggest that FGF10^+^ CAFs are likely to be derived from a mesenchymal cell lineage.

The self-renewal capabilities of fibroblasts are additionally reliant on PDGF signaling. Consequently, PDGFRα is almost constitutively expressed on the progenitor cells of multiple mesenchymal lineages, including fibroblasts [33]. Importantly, scRNAseq has revealed a highly abundant subpopulation of CAFs present during multiple stages of prostate cancer that express PDGFRα, suggesting that their lineage may trace back to progenitor mesenchymal cells [36]. In addition, during tissue repair, fibroblasts transition into a myofibroblast state, a process which is dominantly controlled by the TGFβ pathway [101]. Myofibroblasts derived from mesenchymal cells are reported to highly express TGFβR1/2, which is frequently detected in CAF subpopulations, suggesting that CAF derivation from myofibroblasts involves aberrant TGFβ signaling [57,77,102,103,104].

#### 2.1.3. Bone-Marrow Derived CAFs

Prostate cancer predominately metastasizes to the bone, facilitated by complex interactions between prostate cancer cells and the bone-marrow metastatic niche that are poorly understood [105]. However, bone-marrow cells are also reported to disseminate to the primary tumor site and CAFs have been found to be derived from bone marrow mesenchymal stem cells (BM-MSCs) to support cancer growth and prime tumor cells to metastasize to the bone [56,83]. Moreover, the generation of CAFs from BM-MSCs and their recruitment to the primary tumor is reported to be dependent on TGFβ and SDF-1α (also known as CXC ligand 12, CXCL12) in a mouse model of inflammation-induced gastric cancer [83]. Interestingly, CXCL12 has also been shown to facilitate adhesion of prostate cancer cells expressing CXC receptor 4 (CXCR4) to endothelial cells and ECM proteins via α5β3 integrin to facilitate tumor dissemination [106]. Furthermore, mRNA analysis of the TME from human and mouse prostate cancer bone metastasis specimens showed a high crossover of cells expressing both a bone marrow cell gene signature and high expression of genes strongly associated with myofibroblasts, indicating a CAF population derived from resident bone marrow cells is present within the TME [21].

#### 2.1.4. Endothelial Cell Derived CAFs

Endothelial-to-mesenchymal transition (EndMT) is a process whereby endothelial cells adopt a spindle-like morphology resembling a mesenchymal cell, and has been shown to contribute to the accumulation of CAFs during cancer (reviewed in [107]). Although there has yet to be definitive research showing that CAFs are derived from endothelial cells in prostate cancer, recent studies in other malignancies have shown that EndMT occurs as a result of cancer-derived EVs [108,109]. Indeed, endothelial cells exposed to cancer-derived EVs from murine melanoma, human squamous carcinoma and human breast carcinoma in vitro, all showed upregulation of genes associated with a CAF phenotype and caused increased invasiveness of cancer cell lines [108]. Furthermore, scRNAseq of CAFs in breast cancer has revealed a subpopulation of CAFs that express endothelial markers (e.g., cluster of differentiation 31 (CD31) and nidogen-2) that predominantly localize in proximity to the vasculature, indicating CAFs can originate from endothelial cells [110]. Moreover, Bussard et al., have also suggested a role for TGFβ in converting bone marrow-derived endothelial cells into CAFs [111], however additional work is necessary to determine if this process occurs in prostate cancer.

#### 2.1.5. Epithelial-to-Mesenchymal Transition and CAF Generation

EMT is a process in which epithelial cells alter their phenotype to lose cell–cell adhesion and acquire a mesenchymal phenotype [112]. It is thought that very few CAFs are derived from epithelial cells via EMT as CAFs do not share many molecular markers or genetic alterations with cancer cells, however several studies have highlighted EMT as a possible source of CAFs [113,114]. Analysis of the fibroblast population during kidney fibrosis in mice suggested that approximately 30% were derived from tubular epithelial cells via EMT [115]. Cancer cells that undergo EMT at the invasive front of primary tumors have an increased ability to invade into the local TME and metastasize [116]. Given that EMT is linked to prostate cancer cell dissemination and metastatic spread [117,118,119] and that EMT is associated with the expression of αSMA, FSP1, vimentin and desmin that are commonly detected in CAFs [120], it stands to reason that some of these cells, having adopted a mesenchymal phenotype, could differentiate into CAFs.

Taken together, these data illustrate the numerous potential origins of CAFs within a tumor, including local fibroblasts, myofibroblasts, endothelial cells, epithelial cells, and cells derived from the bone marrow. Of course, there are likely to be even more possible sources of fibroblasts and CAFs than those detailed in this review (e.g., immune cells, adipocytes and adipose tissue-derived mesenchymal stem cells). Thus, future studies delineating the complex origins of CAFs and establishing if the cell-of-origin dictates unique CAF phenotypes and/or functions are therefore paramount.

### 2.2. CAF Activation

The activation of CAFs is commonly linked to the release of factors from damaged/transformed epithelial cells as well as the recruitment of immune cells, similarly to myofibroblast activation [12,13,121]. While TGFβ signaling is perhaps the best characterized stimulus driving CAF activation, CAFs have also been shown to be activated by inflammatory signals, such as IL-1, and genetic alterations within CAFs themselves (discussed below). Changes within the ECM have also been proposed as a potential mechanism for CAF activation (reviewed in [15]), however this is yet to be observed in prostate cancer. For example, an increase in ECM stiffness as well as expression of factors such as fibronectin and collagen type I (COL1) have been implicated in CAF activation [122,123]. Furthermore, anti-cancer therapies have been shown to alter CAF functions and stimulate their activation (discussed in Section 5).

#### 2.2.1. TGFβ-Signaling Causes CAF Activation

TGFβ is a well-studied driver of fibroblast-to-myofibroblast differentiation, and is commonly overexpressed by prostate cancer cells, resulting in constitutive activation of the myofibroblast phenotype through apoptosis evasion and continuous proliferation to form a heterogenous CAF subpopulation [13,48,90,91]. TGFβ is reported to trigger a CAF phenotype in TGFβR1/2-positive myofibroblasts, resulting in the coordination of several physiological processes including ECM remodeling and angiogenesis via SMAD-dependent and -independent pathways [124,125]. Moreover, Wipff and colleagues report that integrin-mediated contraction of myofibroblasts causes the release of TGFβ1 from the ECM during fibrosis, possibly to restrict autocrine generation of myofibroblasts to a stiffened ECM [126]. Despite the plethora of events that can potentiate TGFβ signaling during tumorigenesis (reviewed in [127,128]), TGFβ-mediated CAF activation is speculated to occur in all solid cancers, and is associated with pro-tumorigenic events. For example, co-culture experiments with human DU-145 prostate cancer cells and prostate stromal cells (WPMY-1) have been shown to increase stromal TGFβ signaling and COX-2-dependent ROS production to facilitate cancer cell motility by alleviating estrogen receptor beta (ERβ)-mediated transcriptional regulation of E-cadherin [129]. Furthermore, TGFβ signaling in human prostate fibroblasts has also been shown to induce BPH1 benign prostate epithelial cells to undergo malignant transformation [75] (discussed in Section 4.2.1) and increased oxidative stress in CAFs has been linked to genomic instability in neighboring cancer cells via a bystander effect, potentially contributing to tumor progression [130].

Previous work has shown using QRTPCR that *WNT7A* mRNA transcript levels are increased in a panel of prostate cancer epithelial cell lines in vitro compared to normal/benign prostate epithelial cells, fibroblasts and smooth muscle cells [131]. Interestingly, WNT7A secretion from aggressive breast cancer cells has been shown to stimulate TGFβ signaling, increase CAF abundance and trigger ECM remodeling via non-canonical Wnt signaling to promote tumor invasion in vitro and in vivo, associated with stromal dysplasia and accelerated progression [132]. Furthermore, the capacity for TGFβ to act in an autocrine manner leads to speculation that long term exposure of CAFs to WNT7A may stimulate self-sustained TGFβ signaling to maintain an activated CAF population and facilitate metastasis [132]. Nevertheless, it is important to note that TGFβR1/2-negative subpopulations of CAFs have also been identified, suggesting that TGFβ-independent mechanisms of CAF activation exist, and raises the possibility that activated CAFs can lose their sensitivity to TGFβ during tumor progression [133].

#### 2.2.2. The PI3K/AKT/PTEN Signaling Pathway Mediates CAF Activity

Recent evidence in the literature has indicated phosphoinositide 3 kinase (PI3K) signaling may also contribute to CAF activity. For instance, PI3K hyperactivation in a transgenic mouse model of prostate cancer that harbors an activating mutation in *PIK3CA* (that encodes the PI3K catalytic subunit p110α) has also been shown to exhibit profound stromal remodeling characterized by increased collagen deposition caused by expansion of TGFβ-activated CAFs [134]. Many other signaling pathways that interact with the PI3K and TGFβ signaling cascades have also been implicated in mediating CAF activity, including the mitogen-activated protein kinase (MAPK), WNT, Janus kinase/signal transducer and activator if transcription proteins (JAK/STAT), endothelial growth factor receptor (EGFR) and Hippo signaling cascades (reviewed in [135]); however, to date there has been little research to ascertain whether these signaling pathways regulate CAF activity in prostate cancer.

#### 2.2.3. Notch Signaling Mediated CAF Activation

Notch signaling has also been linked to CAF activation, as the Notch regulator CSL (CBF1, Suppressor of Hairless, Lag-1) is reported to repress fibroblast senescence and CAF activation [136]. Downregulation of *CSL* and p53 in stromal fibroblasts is reported induce fibroblast senescence and the expression of CAF-determinant genes to promote fibroblast-to-CAF differentiation and activation in vitro and in vivo [136]. Moreover, *CSL* expression is down regulated in dermal, oral mucosa, breast, and lung CAFs relative to their respective normal tissue fibroblasts [136]. Conversely, Strell and colleagues have reported that direct cell contact between ductal carcinoma in situ (DCIS) breast cancer cells and peritumoral fibroblasts is sufficient to cause fibroblast-to-CAF differentiation via NOTCH signaling [137]. Notably, loss-of-function studies using CRISPR/Cas9 technology identified epithelial *Jagged1* and fibroblast *Notch2* as key juxtacrine signaling components that can promote PDGFRβ-positive CAF expansion in vivo [137]. Furthermore, co-culturing of osteosarcoma cells with BM-MSCs induced their differentiation into a CAF-like phenotype (i.e., elevated αSMA expression) via Notch-Akt signaling [138]. These findings raise the possibility of a similar mechanism in prostate cancer, which warrants further investigation.

#### 2.2.4. Extracellular Vesicles Regulate CAF Activation

EVs were first described in the 1970s and were originally thought of as “cellular debris” but are now known to be important tools that mediate a number of physiological events, including cell–cell communication. EVs are contained within the cell in endosomal compartments and can fuse to the plasma membrane to release their contents and communicate with target cells [139,140]. EVs have a wide range of signaling functions within the TME, including TGFβ-mediated CAF activation [141,142]. Although the true extent of direct/indirect TGFβ-mediated CAF activation remains to be elucidated, recent evidence suggests that TGFβ paracrine signaling via TGFβ-containing EVs secreted by prostate cancer cells may mediate fibroblast differentiation into myofibroblasts and/or CAFs [142]. Here, EVs isolated from prostate cancer cells were shown to upregulate FGF2 and αSMA in fibroblasts, and EV disruption was found to prevent reactive stroma development in the prostate [142]. Furthermore, tumor-derived EVs harboring *miRNA-21* have been shown to drive the differentiation of hepatic stallate cells into CAFs via activation of protein kinase B (AKT) signaling through phosphatase and tensin homolog (PTEN) downregulation in vitro [143]. Endothelial cells may also differentiate into CAFs upon exposure to tumor-derived EVs, as discussed in 2.1.4 [108,109].

#### 2.2.5. Inflammation-Mediated CAF Activation

Many inflammatory modulators can promote CAF activation during malignant progression [15,144,145]. For instance, Erez and colleagues have shown that the pro-angiogenic, pro-inflammatory activity of skin CAFs is dependent on nuclear factor kappa B (NF-kB) signaling in a transgenic mouse model of squamous cell carcinoma, and that they originate from dermal fibroblasts activated by IL-1β [144]. Prostate CAFs also express pro-inflammatory genes, indicating that prostate CAFs could also be activated in a similar manner.

#### 2.2.6. Senescence and CAF Activation

Senescent fibroblasts differ phenotypically from resting or aged fibroblasts [146], and may additionally secrete factors that promote CAF activation and recruitment [14]. A large degree of overlap between the secretome of senescent fibroblasts and CAFs exists (e.g., IL-6), leading several researchers to classify them as CAFs with low proliferative potential [147]. Although the mechanistic basis for CAF senescence is yet to be unraveled, it is likely to be attributable to physiological and genomic stress. For example, DNA double-stranded breaks in pre-malignant cells can induce IL-6 and activin A secretion that cause CAF infiltration in vitro, but may also cause fibroblasts to enter a senescent state in response to DNA damage caused by high doses of chemotherapy (e.g., doxorubicin) [146,148,149].

## 3. Identification of Prostate CAFs

CAFs are a highly diverse, heterogeneous population of cells derived from a myriad of sources, with no single marker to identify them. Multiple CAF subtypes with unique pro-/anti-tumorigenic functions have been identified using various combinations of protein markers and mRNA signatures (summarized in Table 2). However, current CAF markers are not fibroblast-specific, thus data interpretation is a complex process that involves analysis of cell morphology, localization and the absence of epithelial/immune cell markers [11]. Improving our ability to identify CAF subtypes within the TME using molecular markers could lead to the discovery of new predictive biomarkers that facilitate treatment choices and aid patient care.

CAFs are one of the most abundant stromal cell populations in the prostate cancer (reviewed by [150]) and transcriptomic profiling of the TME has identified a decrease in smooth muscle cell gene expression (e.g., *desmin*, *calponin*, *αSMA*) and increased mRNA expression of CAF-associated genes (e.g., *vimentin*, *PDGFRα/β*, *TGFβ1/2*, *CD90*, *CXCL12*, *FSP1*) relative to normal/benign prostate stroma [26,36,151]. This suggests that the smooth muscle cell population is diminished within the prostate TME, whereas the CAF population is expanded. In support, histopathological analysis and immunohistochemistry (IHC) staining of 40 prostate cancer patient samples revealed that the presence of a reactive stromal (characterized by expansion and thickening of collagen fibers and significant reduction of smooth muscle fibers), correlated with a decrease in αSMA-expressing cells and increased numbers of vimentin-, IGF-1-, MMP-2- and FGF-2-positive cells, that label CAFs [152].

**Table 2:** Summary of mammalian prostate CAF markers, functions and origin.

CAF Subtype	Alternative Nomenclature	Protein and *mRNA* Expression Markers ^1^	Functions	Cell-of-Origin	Reference ^2^
**Identified in prostate cancer**					
**CAFs (generic)** (Human and mouse)	Activated fibroblasts or Activated Myofibroblasts or Tumor-associated fibroblasts (TAFs)	Vimentin, αSMA, TGFβ, AR, TGFβR1/2, FAP, CD90, CD105, COL1A1/2, FSP1, PDPN, CD26, PDGFR-α/β, TNC, ASPN, POSTN, EGF, FGF7/2/10, IGF1, HGF, VEGF, OGN, Fibronectin, FBLN1, CTSK, PARVA, ZEB1, SPARC	ECM remodeling Immune Modulation Angiogenesis Paracrine signaling to prostate cancer cells to promote growth, proliferation and survival.	Resident fibroblasts Endothelial cells Vascular mural cells Epithelial cells Bone marrow-derived cells Mesenchymal stem cells	**[11,25,102,103,153]**
**CD90^+^** (Human)	Reactive stroma fibroblasts or Tumorigenic fibroblasts or Tumor adjacent fibroblasts	CD90, ASPN, *VEGF*, *FGF2*, *PATCH*, *TGFβ*, *IL6*	Tumorigenic	Unknown	**[77,103]**
**CCL2^+^** (Human)	CAF-0	Vimentin, CD90, αSMA, PDPN, LRP1^Low^, *GLRX*, *PKM^Low^*, *CD63^Low^*, *TGFβ*, *CCL2*	CCL2 release to attract TAMs	Unknown (resident fibroblasts predicted)	**[57]**
**CXCL12^+^** (Human)	CAF-1 or SDF1^+^ CAFs	Vimentin, CD90^Low^, αSMA, PDPN, LRP1 *GLRX^Low^*, *PKM^Low^*, *CD63^Low^*, *TGFβ*, *CXCL12*	CXCL12/SDF1α release to attract immune cells and activate AKT signaling via CXCR4 to promote cancer cell growth and survival	Unknown, likely resident fibroblasts	**[57,154]**
**CD105^+^** (Human and Mouse)	Fibroblasts promoting neuroendocrine differentiation or endoglin^+^ CAFs	CD105, αSMA, *TNC*, *SFRP1*	Promotes neuroendocrine differentiation of prostate adenocarcinoma	Unknown	**[6,155]**
**TGFβR2^+^** (Mouse)		TGFβR2, FGF2, *Acta2*, *Tgfβ*, *Vimentin*	Angiogenesis Prostate cancer cell proliferation	Unknown (myofibroblasts predicted)	**[104]**
**TGFβR2^-^** (Human and Mouse)		TGFβR2 negative, αSMA, AR, *Wnt3a*, *CXCL16*, *CXCL1*	Promotes prostate cancer cell adhesion to bone collagen-I fibers to facilitate skeletal metastasis via CXCL1/CXCL16 secretion	Unknown	**[133,156]**
**FGF2^+^** (Human)	Prostate cancer SC-9 cells	*FGF2/7*, TGFβ^high^, VEGF^high^, *COL1A1*, *TNC*, *ACTA2*, *EGF*, *IGF1*	**Predicted functions:** ECM remodeling; collagen Deposition; paracrine TGFβ signaling to prostate cancer cells; angiogenesis	Unknown	**[102]**
**HGF^+^** (Human)	Prostate cancer SC-8 cells	*HGF*, TGFβ, VEGF, *TNC*, *ACTA2*, *EGF*, *FGF7*, *IGF1*,	**Predicted functions:** ECM remodeling and collagen deposition; paracrine TGFβ signaling to prostate cancer cells; angiogenesis.	Unknown	**[102]**
**FGF10^+^** (Mouse)		FGF10	Paracrine FGF10 signaling to prostate cancer cells causes increased AR expression and activated AKT.	Mesenchymal cells	**[100]**
**PDGFR^+^** (Human)		PDGFR	Paracrine PDGFR signaling to prostate cancer cells increased cell motility and invasion.	Resident fibroblasts	**[95]**
**PDGFRβ^+^** (Human)	CAF-S1	*PDGFRβ*, *VIM*, *αSMA*, *CAV1*, *SPARC*, *ETS1*	Cell adhesion and angiogenesis	Unknown (mesenchymal cells predicted)	**[36]**
**PDGFRα^+^** (Human)	CAF-S2	*PDGFRα*, *VIM*, *αSMA*, *CREB3L1*, *PLAGL1*	ECM production and angiogenesis	Unknown (mesenchymal cells predicted)	**[36]**
**Vim^hi^/αSMA^hi^** (Human)	CAF-S3	VIM, FAP, αSMA, TNC, *CAV1*, *MAFB*, *HOXB2*	Fiber contraction to increase ECM stiffness and angiogenesis	Unknown (myeloid cells predicted)	**[25,36,94]**
**Bone marrow** **derived** (Human and Mouse)	Prostate cancer bone metastatic stromal cells	COl1A1, αSMA, VIM, EPHA3, PTN, FSCN1, *FN1*, *TGFβ1*, *TGFβR1/2*, *FGF2*, *CD109*, *PDGFRβ*	Pro-tumorigenic ECM remodeling Skeletal system development Cell adhesion Angiogenesis Wound healing EMT Wnt signaling	Bone marrow resident fibroblasts, bone-marrow-derived mesenchymal stem cells or hematopoietic stem cells	**[21]** Breast cancer: [56] Gastric cancer: [83]
**Identified in other solid cancers**					
**MHCII^+^** (Human and Mouse)	Antigen presenting fibroblasts	MHCII, PDPN, CD74, *COL1A1/2*, *PDPN*, *H2-AB1*, *FAP*, *VIM*	Antigen-specific CD4^+^ T cell activation	Resident fibroblasts	Pancreatic cancer: [157]
**LY6C^+^** (Human and Mouse)	Inflammatory fibroblasts	LY6C, PDPN, IL-1R1, *IL-6*, *COL1A41*, *HAS1*, *CXCL12*, *FAP*, *VIM*	Activates NF-kB and JAK/STAT signaling to promote cancer cell proliferation.	Resident fibroblasts	Pancreatic cancer: [157,158,159]
**Endo180R^+^** (Human and Mouse)	Matrix remodeling CAFs or uPARP^+^ CAFs	Endo180R, PDGFRα, Fibulin-1, *ACTA2*, *FAP*, *Vim*, *Sparc*, *PDGFRβ* *PDPN*	ECM remodeling Collagen internalization and degradation Angiogenesis	Resident fibroblasts	Breast cancer: [110,160,161]
**CD31^+^** (Human and Mouse)	Vascular CAFs	Nidogen-2, CD31, *ACTA2*, *FSP1*, *PDGFRβ*	Angiogenesis	Pericytes or endothelial cells	Breast cancer: [110] Pancreatic cancer: [88]
**Active CD31^+^** (Human and Mouse)	Actively cycling vascular CAFs	Nidogen-2, Ki-67, CD31, *ACTA2*, *PDGFRβ*	Proliferating CD31-CAFs.	Perivascular cells	Breast cancer: [110]
**Developmental** (Human and Mouse)		SCRG1, *Sparc*, *Mia*, *TRL*	Cell differentiation Tissue development and morphogenesis	Predicted: mesenchymal stem cells, or malignant epithelial cells	Breast cancer: [110]
**FSP1^+^** (Mouse)	Fibroblast-like CAFs	FSP-1, VEGFA, TNC	Blood vessel remodeling Apoptosis evasion	Resident fibroblasts	Breast cancer: [93]
**NG2^+^** (Human and Mouse)		NG2, αSMA, PDGFRβ	**Predicted function**: Integrin-dependent PI3K/AKT signaling and chemoresistance	Unknown	Breast and pancreatic cancer: [61,162]
**CD10^+^** (Human)		CD10, GPR77	Stimulates cancer stem cell activity via activation of NF-κB signaling	Unknown	Breast cancer: [163]

^1^ Gray text = expression marker identified but not consistently expressed. ^2^ Bold references = literature specific to prostate cancer. αSMA (*ACTA2)* = alpha smooth muscle actin; AKT (*PBK)*= protein kinase B; AR = androgen receptor; ASPN = asporin; CAV1 = Calveolin-1; CCL2 = C-C motif chemokine ligand 2; CD26 = Dipeptidyl peptidase; CD29 = Integrin β1; CD30 = TNF receptor superfamily member 8; CD31 = Platelet endothelial cell adhesion molecule; CD63 = CD63 molecule; CD90 = Thy-1 pr cluster of differentiation 90; CD105 = Endoglin; COL1A1/2 = collagen type I alpha 1/2 chain; CTSK = cathepsin K; CXCL12 = Stromal cell-derived factor 1 or C-X-C motif chemokine 12; CXCR4 = C-X-C chemokine receptor type 4; ECM = extracellular matrix; EGF = epidermal growth factor; FAP = fibroblast activation protein; FBLN1 = Fibulin 1; FGF2/7/10 = fibroblast growth factor-2/7/10; FSP1 = fibroblast secretory protein 1; GLRX = Glutaredoxin; HGF = hepatocyte growth factor; IGF1 = insulin growth factor-1; PKM = Pyruvate kinase M1/2; LRP1 = Low density lipoprotein receptor-related protein 1; NG2 = Neuron-glial antigen 2; PARVA = Parvin alpha; PDPN = Podoplanin; PDGFR-α/β = platelet-derived growth factor receptor alpha/beta; POSTN = Periostin; pSMAD2/3 = phospho-Mothers against decapentaplegic homolog 2/3; SDF1 = stromal cell-derived factor 1; SPARC = Secreted protein acidic and cysteine rich; STC1 = staneocalcin1; TAMs = tumor-associated macrophages; TGFβ = Transforming growth factor β; TGFβR1/2 = Transforming growth factor β receptor 1/2; TNC = Tenascin; VEGF = vascular endothelial growth factor; ZEB1 = Zinc Finger E-Box Binding Homeobox 1.

The scope of CAF heterogeneity is currently unknown, and combined with the significant overlap and poor specificity of CAF markers, there is a clear unmet need to further stratify CAF subpopulations. To this end, recent studies have begun to subclassify CAF populations using a range of methodologies using bulk/single-cell RNAseq, protein-based assays and lineage tracing [26,36,57,104,152,164,165]. For instance, Vickman and colleagues used scRNAseq to identify six CAF subpopulations in prostate cancer patient specimens (termed CAF 0-5) with distinct functions [57]. However, these six CAF subtypes display shared protein markers (e.g., vimentin/FSP1-positive ‘fibroblast-like’ CAFs and vimentin/αSMA-positive ‘myofibroblast-like’ CAFs) [25,57,94], highlighting the complexity of distinguishing CAF subpopulations. Here, we have surveyed the literature to outline recent developments in the methodologies used to identify CAF subpopulations in prostate cancer to provide a comprehensive, unbiased overview of CAF subtype classification approaches and help inform the selection process of CAF markers for future studies.

### 3.1. RNA-Based Identification of CAF Subpopulations

Advances in transcriptomic profiling have dramatically improved our molecular understanding of prostate cancer and as introduced above, scRNAseq of isolated human fibroblasts from localized primary prostate tumors has led to the identification of six prostate CAF subpopulations (termed CAF-0-5) with distinct immune modulatory functions, two of which have been extensively characterized (CAF-0/1) [57]. Of note, each of the six subpopulations express high levels of vimentin (*VIM*) mRNA and moderately increased levels of fibroblast activation protein (*FAP*) mRNA in comparison to normal fibroblasts [57]. Prominent differences in podoplanin (PDPN), cluster of differentiation 26 (CD26), αSMA, and CD90 transcript levels were also detected when comparing CAF0-5 subtypes, and relative to matched normal fibroblasts [57]. Likewise, tumor promoting chemokines, such as CC ligand 2 (CCL2) and CXCL12 were differentially expressed within these six CAF subpopulations, correlating with diverse immunomodulatory functions; CCL2-positive CAFs (CAF-0s) were found to attract tumor-associated macrophages, while CXCL12-positive CAFs (CAF-1s) could attract other immune cells (e.g., mast cells, innate lymphoid cells, eosinophils), both of which ultimately contribute to development of an immunosuppressed TME in the prostate [57]. Interestingly, both CAF-0 and CAF-1 populations express high levels of CD90, a glycophosphatidylinositol cell adhesion molecule anchored on the cell surface. CD90 is reported to promote tumorigenesis through regulation of the immune system and cytokine/TGFβ secretion, activation of hedgehog (Hh) signaling and angiogenesis [77,103]. Additionally, co-expression of CD90 and CXCL12 in primary CAFs isolated from prostate tumors with Gleason scores of 3 + 4 or 4 + 4 positively correlates with induction of TGFβ-dependent pro-tumorigenic CXCR4 signaling in human BPH1 cells in co-culture assays or with conditioned media (CM) from CD90^High^ CAFs, although the magnitude of the TGFβ-dependency remains to established [77]. Remarkably, CD90^+^ CXCL12^+^ CAFs were found to protect BPH-1 cells against hydrogen peroxide induced apoptosis in vitro, suggesting that CD90^+^ CAFs may promote prostate epithelial cell survival [77]. CD90 upregulation is also reported to serve as a potential biomarker for the emergence of a reactive stroma associated with high CAF abundance, and has been identified in both human primary prostate tumors and lymph node metastases [166].

An independent scRNAseq study has also classified CAFs into three subtypes (termed S1-3) with distinct mRNA signatures (outlined in Table 2), where all three populations express *VIM* and *ACTA2* mRNA transcripts [36]. Notably, transcription factor and gene enrichment analysis suggests that while each of the three CAF subtypes identified regulate angiogenesis, distinct functional differences exist, with S1 showing enrichment of genes controling cell adhesion, S2 displayed upregulation of genes involved in ECM production, and S3 exhibited an increase in fiber contraction genes [36]. Interestingly, S1 and S3 also show increased transcription of the oncogenes *ETS1* and *HOXB2*, respectively [36]. Furthermore, it has been suggested that the presence or absence of key surface markers on CAFs, such as TGFβR2 and CD90, may provide a more simplistic and broader approach to characterization [77].

Analysis of mouse prostate CAFs by scRNAseq, to the best of our knowledge, is yet to be undertaken. However, scRNAseq of transgenic mouse mammary tumor fibroblasts has identified four murine mammary CAF subtypes: vascular CAFs (CD31-expressing CAFs), actively cycling vascular CAFs (active CD31-expressing CAFs), developmental CAFs and matrix-remodeling CAFs (Endo180R-expressing CAFs) [161]. It will be interesting for future work to establish if prostate CAFs subcategorize in a similar manner and/or frequency, as different tumor types have been shown to display CAF subpopulations with similar markers, yet differing abundance [14,161]. For instance, immunofluorescent staining of CAFs identified by their morphology shows that co-expression of FSP1 and αSMA occurs in 10.9% of the total CAF population in the 4T1 murine breast carcinoma model, compared to 43.5% in the Rip1Tag2 pancreatic tumor model [61]. The abundance of distinct CAF subpopulations may also provide insight into the predominant CAF cell-of-origin. For example, melanoma patients often present with a high population of PDGFRα-positive CAFs, suggesting that resident dermal fibroblasts transitioned into CAFs [14,96].

Combined tissue microarray (TMA) and QRTPCR analysis of matched normal prostate fibroblasts and CAFs from patients with a Gleason score of 6–9 (*n* = 17) has also identified several proteins and genes that are differentially expressed in CAFs relative to normal fibroblasts [26]. *MAGT1*, *MAK3K1*, *PHLDB2*, *RNY3*, and *SCARB1* mRNA transcripts were significantly upregulated in CAFs, whereas *SNAPC2* and *FSP1* were downregulated, suggesting that aberrant TNF, NF-kB, TGFβ and MAPK signaling occurs in CAFs to regulate processes such as cell survival, proliferation and migration [26]. Consistent with the literature indicating CAFs promote cell survival and angiogenesis, *TGFβ*, *PDFGRβ* and *CXCL12* mRNA transcripts were also found to be elevated in prostate CAFs [26]. Furthermore, microarray gene expression analysis of laser captured Gleason grade 3 prostate reactive stroma relative to matched benign stroma (*n* = 17) revealed alterations in several oncogenic processes, including ECM remodeling, angiogenesis and neurogenesis [167]. Gene expression profiling of isolated lines of prostate cancer patient matched pairs of normal fibroblasts and CAFs has also revealed enrichment of 671 genes highly associated with prostate morphogenesis and reduced expression of 356 genes highly associated with cell cycle in CAFs relative to normal fibroblasts [103], highlighting their diverse transcriptomes and the possibility that more specific fibroblast/CAF mRNA markers may be identified in the future.

### 3.2. Protein-Based Assays to Identify CAFs

Protein markers have been instrumental in detecting prostate CAF subpopulations. These include the well characterized vimentin, αSMA and a range of growth factors, enzymes, chemokines, and signaling molecules known to facilitate cancer progression (summarized in Table 2). In addition, FGF2-, hepatocyte growth factor- (HGF), FGF7-, FGF10-, AR-, MMP-11-, heat shock protein family A member 1A (HSPA1α)-, endoglin (CD105)-, CXCL12-, CCL2-, endo180- and PDGFR-expressing CAFs can also independently positively correlate with disease stage and stromal responses [57,95,100,102,161]. For example, the presence of FGF7-positive CAFs isolated from prostate cancer biopsies is linked to localized prostate cancer, whereas MMP-11, AR and HSPA1α positively correlate with metastatic CRPC, established by QRTPCR and IHC [168]. Several researchers have also isolated prostate CD105-expressing CAFs and demonstrated their pro-tumorigenic action in promoting neuroendocrine differentiation during prostate cancer (Table 2) [6,155]. Indeed, primary human CD105^+^ CAFs show increased expression of secreted frizzled related protein 1 (SFRP1) compared to normal CAF lines, and have been shown to promote neuroendocrine differentiation in prostate cancer cells in vitro [6]. Nevertheless, it is widely acknowledged that CAF protein markers used to date are not CAF specific, such as vimentin and αSMA that are expressed in malignant epithelial cells and a number of other stromal cell types residing in the TME (reviewed in [11]). Additional non-specific CAF markers include PDGFRα/β, which is also expressed in normal fibroblasts, smooth muscle cells and glial cells [169], CD90, which is also expressed in stem cells, neurons and activated endothelial cells [170,171] and TGFβR1/2 that is expressed in cancer cells [11].

Recently, the collagen receptor endo180 (also known as uPARP) has also emerged as a novel marker of CAFs in breast and colorectal cancers that secrete pro-tumorigenic matrix components and matrix-modifying enzymes, such as MMPs and collagenases [161,172,173,174]. Endo180 activity in normal mesenchymal cells is linked to collagen internalization and degradation, angiogenesis, cell chemotaxis and migration, which could facilitate cancer growth and progression (reviewed by [174]). Spheroid cultures of CAFs transfected with Endo180 (*Mrc2*) siRNAs showed reduced viability and altered contractility; and systemic deletion of *Mrc2* significantly reduced tumor burden and metastatic progression of 4T1 mouse mammary carcinoma cells in vivo [161]. High endo180 receptor expression in mammary and colorectal CAFs also correlates with an elevated fibroblast TGFβ response signature that is associated with poor outcome in several cancers, including prostate cancer [161,172,173,174]. Nevertheless, the presence and functional importance of endo180^+^ CAFs during prostate tumor onset, progression and therapeutic resistance remains to be determined, and could prove to hold predictive value and/or present a valuable stromal therapeutic target.

Taken together, protein and RNA based analysis of CAFs has provided valuable insights into the plethora of CAF subpopulations found within the prostate TME. Nonetheless, substantial differences in the markers/approaches employed exist, making it difficult to compare experiments and determining the CAF cell-of-origin remains a significant challenge. Analysis of a combination of CAF markers together with cell morphology and localization is currently essential to avoid false positives and to improve accuracy of identifying CAF subpopulations to better understand the significance of their frequency, distribution and inter/intra-tumoral heterogeneity during prostate tumor formation, progression and drug-resistance. In the advent of single cell resolution proteomic and transcriptomic digital spatial profiling, there is hope that some of these complexities may be unraveled and a more accurate classification system established. Moreover, future research is needed to determine the clinical relevance of each CAF population within primary and metastatic prostate tumors, as well as a deeper understanding of prostate CAF populations/functions in mouse models to improve our interpretation of preclinical trials and increase clinical translatability. How CAF subtypes differ according to the mouse genetic background also needs to be explored, along with determining the clinical relevance of mouse CAFs. Consideration of CAF immunomodulatory function in immunocompromised mice vs. immune proficient mice will also be important, particularly when exploring immunotherapies in vivo [57]. Lineage tracing studies may also offer new information into CAF generation, recruitment and activation, and could lead to the identification of new CAF-targeted therapies.

## 4. Prostate Cancer-Associated Fibroblast Functions

CAFs have been shown to mediate a myriad of functions to promote or suppress tumor formation and progression [11,13,14]. To address the diverse and complex roles of CAF subpopulations during prostate tumorigenesis, we have reviewed the recent advances in the field below to emphasize their distinct functions and highlight critical signaling events and cell–cell communication mechanisms involved.

### 4.1. CAFs Remodel the Extracellular Matrix to Facilitate Tumor Growth and Progression

Maintenance of the ECM is essential for normal adult prostate tissue homeostasis and is dependent on several factors, including the correct deposition of ECM components. The generation, recruitment and activation of CAFs results in ECM remodeling via CAF-mediated production of ECM components such as collagens, TNC, hyaluronan, fibronectin and MMPs, which disrupts normal tissue architecture and promotes tumorigenesis [14,25,175,176,177].

#### 4.1.1. CAF-Mediated Collagen Deposition

Collagen constitutes the primary structural element within the ECM and its deposition is essential for supporting tissue development, providing tensile strength, regulating cell adhesions and enabling chemotaxis and migration [178]. High collagen deposition by CAFs (especially type I and II) causes matrix stiffening and increased ECM thickening caused by reorientation and crosslinking of collagen fibers and elastins to produce larger, more rigid fibrils [179,180,181]. ECM stiffening and thickening has been shown to facilitate prostate cancer EMT and invasion and encourage immune cell infiltration, respectively [182,183,184,185]. Increased ECM thickness is also reported to increase the activity of focal adhesion kinase (FAK), Rac, extracellular signal-regulated kinase (ERK) and yes-associated protein/transcription co-activator with PDZ-binding motif (YAP/TAZ) signaling cascades, which facilitate tumor growth, progression and invasion [186,187,188]. Additionally, increased ECM thickness and stiffness has been shown to intensify interstitial pressure within the TME, which is associated with hypoxia owing to reduced blood flow and can impair intravenous drug delivery (reviewed by [189]). Indeed, hypoxic regions in PC-3 prostate cancer xenograft tumors display reduced COL1 fiber density and more COL1 fiber structural alterations relative to normoxic regions, associated with increased lysyl oxidase (*LOX*) mRNA expression and reduced MMP gene transcripts that could impair cancer cell dissemination [182]. Furthermore, Penet and colleagues identified distinct significant differences in COL1 fiber patterns, as well as an increase in COL1 deposition and CAF abundance in clinical metastatic prostate cancer specimens [185]. A strong association between COL1 levels and number of bone marrow metastases in prostate cancer patients has also been reported, suggesting a possible role for COL1 as a prognostic biomarker [183]. Taken together, these findings illustrate that dysregulated collagen deposition by CAFs within the prostate TME can promote metastatic progression and predict for worse outcome.

#### 4.1.2. Tenascin C (TNC)

TNC is a matricellular glycoprotein that is highly expressed during embryonic development, tissue repair and oncogenesis, and can mediate cell signaling, proliferation and migration (reviewed by [190]). During mouse prostate development, TNC has been identified as a driver of epithelial differentiation, raising the possibility that TNC may also play a role in EMT during tumorigenesis [191], and TNC expression has also been detected in CAFs [25,59,192,193] (Table 2). In BPH and prostate cancer, myofibroblasts and vimetin^+^/αSMA^+^ CAFs have been shown to secrete TNC in response to TGFβ signaling, epithelial cell-derived-IL-8 and/or tensile strain within the ECM [25,59,192,193]. Accordingly, TNC is a common hallmark of reactive stroma in the prostate. In vitro studies have also shown TNC can promote migration of myofibroblasts during wound repair and can stimulate invasion of colon cancer cells by inducing a change to an elongated, migratory morphology, involving increased Rac activity and downregulation of RhoA [194,195]. TNC is also reported to be significantly expressed alongside FSP1, αSMA and vimentin, and positively correlates with poor survival in patients with prostate cancer [196].

#### 4.1.3. Hyaluronan

The glycosaminoglycan hyaluronan constitutes a major component of the ECM and is implicated in cell division and migration during embryogenesis, inflammation and wound repair [197]. In prostate cancer, TMA analysis has revealed that high hyaluronan IHC staining positively correlates with a high Gleason score, larger tumor volume and worse clinical outcome [176]. Furthermore, intraprostatic injection of hyaluronan has been shown to significantly increase tumor growth in a rat model of prostate cancer [176]. CAFs have also been shown to secrete high levels of hyaluronan synthases that control the biosynthesis of hyaluronan in oral squamous cell carcinoma, suggesting that elevated hyaluronan production is likely to be a common event in other epithelial cancers [198].

#### 4.1.4. Fibronectin

Fibronectin is a fibrous protein involved in organization of the ECM and mediation of cell attachments [46]. However, fibronectin deposition has also been linked to invasive tumor progression, owing to its role in mediating cell migration during development [199,200]. Intriguingly, high fibronectin networks assembled by primary human prostate CAFs in vitro have been shown to interact with integrin-α5β1 on prostate cancer cells to produce migration paths to facilitate and guide their migration [175]. A similar interaction was also shown between PDGFRβ^+^/αSMA^+^ CAF-derived fibronectin and integrin-αv/β3 positive colon cancer cells, where absence of fibronectin completely abrogated tumor cell invasion [201]. These data suggest that agents targeting fibronectin may prove to be effective therapeutics by reducing invasive potential.

#### 4.1.5. Matrix Metalloproteinases (MMPs)

MMPs are multifunctional proteases secreted by fibroblasts, CAFs and epithelial cells that can proteolytically cleave ECM components, releasing bioactive fragments and proteins to degrade the ECM [202]. MMPs can also regulate extracellular tissue signaling networks to mediate events such as angiogenesis, wound healing, cell migration/invasion and immune response (reviewed in [202]). In prostate cancer, CAFs and prostate cancer cells have both been shown to produce MMPs to increase invasive potential [168,202,203,204]. Interestingly, this has been linked to loss of the Wnt antagonist dikkopf-3 (DKK3) in both epithelial and stromal cells [205,206]. Secreted DKK3 can suppress TGFβ signaling and is also thought to be associated with MMP-2 and MMP-9 secretion [205,206], adding an additional layer of complexity to TGFβ-mediated CAF differentiation and activation. Moreover, DKK3 depletion in WPMY-1 prostate stromal cells is reported to increase TGF-β signaling activity and extracellular matrix protein 1 (ECM-1) secretion, and stromal cell-conditioned media from DKK3-deficient WPMY-1 cells was found to inhibit prostate cancer cell invasion [205]. Several MMPs have been implicated in prostate cancer growth and progression, for example increased MMP-2 expression is associated with advanced disease and a highly reactive stroma [152,207]. MMP-2 has also been shown to be produced by a number of CAF subpopulations, including endo180^+^ CAFs in breast cancer [152,161,207]. Although exploration of MMP inhibitors for the treatment of prostate cancer has commenced, to the best of our knowledge clinical data reporting their efficacy remains to be disclosed [202].

In corroboration, these findings indicate that CAFs coordinate numerous ECM components to remodel the ECM and influence tumor growth, thus presenting an attractive route for therapeutic intervention. However, preclinical and clinical research in this field is currently limited, and further work is needed to determine if targeting CAF-driven pro-tumorigenic ECM remodeling processes is effective against prostate cancer.

### 4.2. The CAF Secretome Contributes to Prostate Cancer Growth and Metastatic Progression

CAFs mediate numerous physiological processes that can contribute to tumorigenesis such as angiogenesis, stemness, immune response and metastasis. To achieve this, the CAF secretome comprises a range of signaling molecules, including chemokines (e.g., CXCL12, CCL7), cytokines (e.g., ILs, TGFβ), proteins (e.g., periostin, TNC, PTEN, HSP90, sonic hedgehog (SHH), YAP1), growth and angiogenic factors (e.g., FGF2/7/10, HGF, IGF1, PDGF, TGFβ, VEGF, SDF1) and extracellular vesicles whose cargo can regulate a range of cell signaling events (e.g., DNA, mRNA, miRNA and proteins) [11,14,74,151]. Here, we review the diverse cellular and physiological processes mediated by the CAF secretome that can promote prostate cancer growth and progression.

#### 4.2.1. CAF-Mediated Oncogenic Signaling

While TGFβ can induce a CAF phenotype, it is also secreted by CAFs and positively correlates with CRPC growth in patients and mouse models [25,126,129,208]. CAF-mediated TGFβ autocrine signaling is crucial for self-regulation, whereas TGFβ paracrine signaling enables cross-talk between CAFs and neighboring cells (e.g., via the release of EVs) [132,142]. Importantly, TGFβ can serve as a central node to mediate CAF function by cross-regulating multiple signaling pathways to support tumor growth, including PI3K/AKT, CXCL12, FGF, and Wnt/β-catenin cascades [76,132,134,205,206]. In addition, ROS-producing NADPH oxidase (Nox4) is reported to be essential for TGFβ-mediated activation of prostate fibroblasts to a CAF-like phenotype, and inhibiting stromal Nox4 was found to suppress prostate cancer cell proliferation and migration induced by TGFβ1-activated prostate fibroblast conditioned media [209,210].

Given that analysis of clinical specimens has revealed that TGFβR2 loss is evident in 69% of human prostate cancer-associated stroma and 15% of benign prostate tissue stroma [133], it is likely that not all CAFs are regulated by TGFβ signaling. Moreover, loss of TGFβR2 function in half of the stromal population in a tissue recombination study caused malignant transformation of BPH1 prostate epithelial cells, induced expression of myofibroblast differentiation markers and augmented TGFβ and AKT signaling [75]. These findings indicate that heterogeneous TGFRβR2 expression in fibroblast subpopulations can promote epithelial cells to undergo malignant transformation to facilitate tumor growth. CAFs isolated from *Tgfβr2* knockout mice have also been shown to increase tumor growth and proliferation in LNCaP human prostate cancer recombinants in a Wnt3a-dependent manner [133], indicating TGFβR2-deficient CAFs may contribute to prostate cancer growth in patients. In support, loss of stromal TGFβR2 in *FSPCre^+^ Tgfbr2^fl/fl^* transgenic mice has been shown to cause prostate intraepithelial neoplasia (PIN) within six weeks of age, and these mice did not survive beyond seven weeks [133]. However, additional work in prostate tissue recombination allografts in the renal capsule revealed that stromal loss of *Tgfbr2* in mice can facilitate tumor progression to adenocarcinoma [133]. Conversely, Yang and colleagues have shown that tumor burden is reduced when LNCaP prostate cancer cells are co-inoculated with either TGFβR2-null or dominant negative SMAD3 stromal cells into mice, associated with a reduction in micro-vessel density, depletion of FGF2-positive cells and attenuated TGFβ signaling in the stroma [104]. Thus, it will be important for future work to determine the predictive value of stromal TGFβR2 in prostate cancer, and to delineate the molecular mechanisms underpinning tumor progression in the context of TGFβR2 stromal loss.

Several CAFs identified in the literature have been shown to express high levels of FGFs (predominantly FGF2, FGF7 and FGF10) [100,102,104,211,212] (Table 2). Similarly to TGFβ, FGFs secreted by CAFs have autocrine/paracrine tumor growth stimulating effects [100,102,104,211,212]. Moreover, stromal *Fgf2* depletion in the TRAMP mouse model of neuroendocrine prostate cancer has been shown to increase survival and decrease tumor growth and metastasis compared to wild-type TRAMP mice [212]. The FGF-FGFR signaling axis has many downstream effector cascades, including RAS/MAPK, PI3K/AKT/mTOR and JAK/STAT signaling pathways [213,214], thus providing potential mechanisms whereby CAF-mediated activation of FGF signaling in prostate cancer cells could promote tumor growth.

Interestingly, Eph-Ephrin signaling that mediates cell migration and cell–cell adhesion has also been shown to mediate CAF-tumor cell cross-talk. For instance, elevated levels of EphB3 and EphB4 receptors on prostate cancer cells has been shown to impair contact-inhibition locomotion in fibroblast co-cultures assays [215]. EphrinB2 ligand expressed on prostate CAFs can interact with the EphB3/4 receptors on the surface of prostate cancer cells to activate Cdc42 signaling and increase the invasive potential of PC-3 cells in vitro [215]. Furthermore, inhibition of EphA2 receptor via synthetic or natural agonistic ligands significantly reduced metastasis in the PC-3 orthotopic xenograft mouse model, while EphA7 overexpression in PC-3 and DU-145 prostate cancer cells has been shown to decrease tumor volume and increased apoptosis [216,217]. These studies illustrate the complex nature of Eph-Ephrin signaling during prostate cancer growth and progression and emphasize the need to better understand how this pathway coordinates tumor–stroma interactions, which could reveal new therapeutic avenues and/or biomarkers.

EVs released by CAFs have also been shown to contribute to tumorigenesis. For example, CAF-secreted EVs containing non-coding RNAs (e.g., microRNA-409) have been found to correlate with a high Gleason score and metastatic disease [218]. CAF-derived EVs can also inhibit translation of tumor-suppressor genes (e.g., RSU-1, Ras suppressor 1 and STAG2, stromal antigen 2) and promote EMT via increased expression of vimentin and β2-M [218]. Furthermore, EVs originating from CAFs have been shown to contain mitochondrial DNA that can restore metabolic activity of cancer stem cells to promote development of hormone therapy-resistant breast cancer [219]. On the other hand, EVs derived from prostate cancer cells have been shown to drive the differentiation of normal fibroblasts into CAFs (discussed in Section 2.2.4), highlighting that EVs mediate tumor–stroma cross-talk in a bidirectional fashion. Accordingly, significant research efforts are underway to develop our molecular understanding of EV signaling between the stromal and epithelial compartments during prostate cancer, and to determine the predictive value of EV cargo [220,221,222,223,224,225].

#### 4.2.2. CAF Regulation of Angiogenesis

Angiogenesis refers to the formation of new blood vessels to support tumor growth by supplying oxygen, nutrients and signaling molecules, and accumulating evidence suggests that this process can be regulated by the CAF secretome in prostate cancer [11,13,14,51]. For instance, IL-6 secretion by CAFs has been shown to facilitate angiogenesis in vitro through the stimulation of VEGF secretion by prostate cancer cells, which is also known to facilitate ADT resistance [226,227,228]. Indeed, CAF-derived IL-6 can induce VEGF secretion in established prostate cancer cells via PI3K/AKT signaling, and is independent of AR activation [227]. In vitro studies have also revealed IL-6 released by CAFs causes endothelial cell migration [229], which could result in increased vasculogenesis and angiogenesis to facilitate increased tumor growth and dissemination. Interestingly, combined engraftment of LNCaP human prostate cancer cells and vimentin^+^/αSMA^+^ CAFs in mice has also been shown to stimulate angiogenesis during early tumor progression [25].

Multiple CAF subpopulations have been shown to express VEGF, a potent angiogenesis inducing growth factor (Table 2) [77,93,102,103]. QRTPCR analysis of FACS sorted human prostate cancer CD90^+^ CAFs revealed a significant upregulation of *VEGFA* mRNA expression relative to normal fibroblasts, while Tag profiling of human prostate cancer CAFs reported an increase in *VEGFD* expression in CD90-negative CAFs [77,103], indicating that different subpopulations of CAFs may induce angiogenesis via upregulation of distinct growth factors. Interestingly, human prostate cancer primary CAFs co-cultured with androgen-sensitive prostate cancer cell lines showed high protein levels of CAF-derived VEGF, however this effect was not observed with androgen-insensitive prostate cancer cells [102]. CAFs may also facilitate the formation of a pro-angiogenic microenvironment at during metastasis at the site of colonization [93].

Conversely, CAFs have also been shown to suppress angiogenesis. For instance, depletion of stromal content, including CAFs, in a transgenic pancreatic cancer mouse model harboring a *Shh* loss-of-function mutation is reported to cause increased cancer proliferation and angiogenesis [230]. These findings suggest CAFs play diverse roles that may be dependent on the disease stage, tissue type and/or CAF subtype. Thus, it will be important for future work to explore how CAFs mediate angiogenesis to better understand how they can be effectively targeted therapeutically.

#### 4.2.3. CAFs Mediate Metastatic Potential

Metastatic prostate cancer presents a major clinical challenge, highlighted by the fact that the 5-year survival rate for patients diagnosed with metastatic prostate cancer is currently <30% (reviewed in [231]). Although the role of CAFs during prostate cancer metastasis remains to be fully elucidated, several studies have indicated that targeting CAFs may have an anti-metastatic effect. For instance, depletion of FSP1^+^ CAFs can reduce metastatic burden and distribution in the syngeneic 4T1 metastatic breast cancer mouse model, correlating with reduced VEGFA and TNC secretion, decreased angiogenesis, and increased tumor cell death [93]. Moreover, CAF depletion has been found to reduce survival of metastatic lung cancer cells in mice whereas co-inoculation of cancer cells and CAFs increased survival [232,233], suggesting that CAFs can disseminate into the circulation and travel to distant sites to prime the metastatic niche and protect circulating cancer cells. Moreover, circulating stromal cells are regarded as CAF-like cells derived from endothelial or epithelial cells that have undergone EndMT or EMT, respectively [88,234]. These data support Paget’s ‘seed and soil’ hypothesis [235], however further work is needed to better understand CAF dissemination and whether this is a tissue specific event or not. Interestingly, while bone marrow-derived stem cells have been shown to transition into CAFs in mouse models of inflammation-induced gastric cancer to promote tumor growth via expression of IL-6, Wnt5a and BMP4 [83], prostate cancer cell secretion of BMP4 has recently been shown to instruct endothelial cells within the TME to undergo transition into osteoblasts in osteogenic prostate cancer xenograft models, encouraging bone matrix mineralization through BMP4-driven p-SMAD1/Notch and GSK3β/β-catenin/OSX signaling [236]. Taken together these findings highlight the complexity of cell plasticity within the TME and the challenges faced in monitoring and targeting CAF subpopulations throughout primary tumor evolution and at distinct metastatic sites.

TGFβR2-expressing and TGFβR2-negative CAFs have also been shown to facilitate metastatic progression, and have been observed in both primary and metastatic prostate cancer clinical specimens [104,156,237]. Mechanistically, TGFβR2-negative prostate CAFs were found to increase CXCL1, CXCL16 and CXCL5 expression and facilitate prostate cancer cell adhesion to bone COL1 fibers to promote skeletal metastasis in C4-2B xenografts [156]. In addition, depletion of TGFβ2 in FSP1^+^ CAFs has been shown to induce squamous cell carcinoma in the murine forestomach, associated with the upregulation of bone morphogenetic protein 7 (BMP7), SMAD1/5/8 and HGF [238]. Interestingly, the presence of FSP1-positive CAFs has also been linked to a poor prognosis in prostate cancer [239,240], indicating FSP1^+^ CAFs may play a pro-metastatic role. Indeed, O’Connell et al. showed that in breast cancer, FSP1^+^ CAFs produce VEGFA and TNC, which can promote remodeling of blood vessels and apoptosis evasion, respectively [93,196,241]. Notably, TNC expression that is normally absent in mature adult bone has been shown to be expressed during prostate cancer bone metastasis, possibly facilitating prostate cancer cell homing to the bone marrow niche [242]. Moreover, bone marrow-derived CAFs, thought to originate from either BM-MSCs or resident fibroblasts, have been found to enhance primary tumor growth and metastatic potential in prostate, breast and gastric cancer xenografts and a mouse model of inflammation-induced gastric cancer [13,20,21,56,83] (discussed in Section 2.1.3).

#### 4.2.4. CAFs and TME Immunomodulation

Multiple studies spanning several cancer types (including prostate, breast and colon) have reported a strong correlation between the CAF secretome and the creation of an immunosuppressive microenvironment to aid tumor growth [12,243,244,245]. This stems from the fact that CAF secretion of proinflammatory factors into the TME can attract tumor suppressive immune cells [14,57], in addition to the metabolic by-products of tumor cells and TME acidity [246]. Indeed, in vitro studies have shown a reciprocal relationship between primary human prostate cancer CAFs and inflammatory macrophages, whereby CAFs can stimulate monocyte recruitment and promote an M2 macrophage-like phenotype via CAF-derived secretion of CXCL12, and M2-like macrophages were able to elicit activation of normal human prostate fibroblasts via upregulation of αSMA [247].

Nevertheless, prostate cancer is generally regarded as an immunologically cold malignancy, and several studies have suggested this may reflect CAF enrichment [244,248]. A positive relationship between CAF abundance and an immunologically cold TME has been observed using transcriptomics, flow cytometry and histopathological analysis in mouse mammary carcinoma [245]. Moreover, CAF abundance in breast cancer mouse models, produced via transplantation of 4T07 or D2A1 cells into the mammary fat pad of BALB/c or NSG mice, was recently shown to positively correlate with immune checkpoint blockade resistance via suppression of CD8^+^ T cell infiltration [245], suggesting that the presence of CAFs has implications for immunotherapy sensitivity. In support of this notion, a reduction in the number of αSMA-expressing CAFs in murine mammary tumors upon endo180 genetic deletion is reported to inversely correlate with CD8^+^ T cell infiltration, leading to enhanced immune checkpoint inhibitor efficacy in vivo [245]. Accordingly, prostate tumors with high CD8^+^ T-cell infiltration may also secrete TGFβ, resulting in the activation of CAFs [249,250]. These findings indicate that immune cell TGFβ-mediated activation/recruitment of CAFs may subsequently cause tumor immune evasion through the depletion of immune suppressive CD8^+^ regulatory T cells to further promote tumor growth. Indeed, several groups have identified novel ECM-related and CAF-associated gene signatures that can predict resistance to immune checkpoint blockade (ICB) (reviewed in [251]). TGFβ has been well-characterized as an immunosuppressive cytokine via several mechanisms, including; (i) reducing Ca^2+^ influx to suppress transcription of *NFATc*, *T-bet* and *GATA-3* that reduces T-cell differentiation and proliferation, (ii) upregulating *FOXP3* together with IL-2 in naive CD4^+^ T cells, resulting in their conversion to regulatory T (Treg) cells and (iii) reducing dendritic cell antigen presentation by suppressing the expression of major histocompatibility complex 2 (MHCII) (reviewed in [251]).

In addition, exploration of the immune modulatory functions of CAFs has also revealed that certain CAF subpopulations can modulate CD4^+^ T-cells and macrophage responses [57,157,247,251]. Notably, newly identified MHCII and CD74 expressing CAFs in pancreatic ductal adenocarcinoma (PDAC) termed “antigen presenting CAFs” are reported to activate CD4^+^ T cells in an antigen-specific manner, but do not appear to induce T-cell proliferation [157]. Activation of JAK/STAT in fibroblasts via TGFβ and IL1 secreted by PDAC cells in vitro has also been shown to induce fibroblast differentiation into an inflammatory CAF phenotype (LY6C^+^ CAFs), correlating with upregulated transcription of genes that facilitate the recruitment of inflammatory cells, such as *Il-6*, *Cxcl12* and peptidase inhibitor 16 (*Pi16*) [157,158,159]. Furthermore, in vitro assays have shown CCL2-secreting CAFs (or CAF-0s) attract tumor-associated THP-1 macrophages, while CXCL12-secreting CAFs (or CAF-1s) can attract monocytes and trigger their differentiation into M2-like macrophages [57,247]. Hence, both CAF-0 and CAF-1 subpopulations may facilitate an immunosuppressive TME in the prostate [57].

Controversially, CAFs have also been shown to harbor anti-tumorigenic activity. Although CAF tumor suppressive functions have not been well-characterized to date, CAF anti-cancer functions are considered to occur early during tumorigenesis and involve the creation of an immune permissive TME [13]. This view relates to the CAF secretome containing factors such as IL-10, TGFβ, IFNγ and IL-6 that are known to recruit macrophages, natural killer cells and CD3^+^ T lymphocytes during tumor initiation to promote an anti-tumor immune response (reviewed by [12,13]). In support, depletion of αSMA-positive CAFs in a transgenic mouse model of pancreatic cancer (*Ptfla^cre/+^*; *LSL-Kras^G12D/+^*; *Tgfβr2^flox/flox^*) during either non-invasive precursor or PDAC stages of oncogenesis has been shown to enhance the Treg cell population and intra-tumoral hypoxia, leading to a more invasive tumor phenotype [184].

In summary, CAF subpopulations mediate diverse, spatiotemporal functions that involve the integration of numerous signaling networks within the TME and extensive tumor–stroma interactions that together can promote tumor growth and metastatic progression. However, it is crucial that research continues in this field to provide the necessary information required to develop effective anti-cancer CAF-directed therapies and/or predictive CAF biomarkers, and enable us to take advantage of the beneficial effects of CAF-mediated anti-tumorigenic functions during the early stages of malignancy.

## 5. CAFs Contribute to Therapeutic Resistance in Prostate Cancer

### 5.1. CAFs and Therapeutic Resistance to Androgen/AR-Directed Therapy

**Figure 1:**
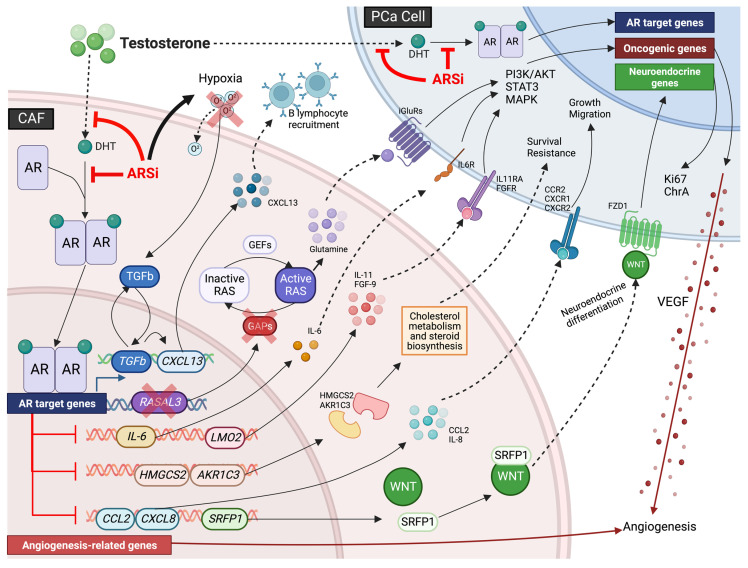
Overview of potential CAF-mediated resistance mechanisms to androgen receptor signaling inhibitors (ARSi). To overcome ARSi, (e.g., enzalutamide treatment), CAFs may coordinate a number of processes including; hypoxia-induced TGFβ-CXCL13 signaling to increase B-cell recruitment, overactivation of RAS resulting in glutamine mediated oncogenic signaling, augmented SRFP1/WNT signaling to increase TNC expression and induce neuroendocrine differentiation, enhanced cholesterol metabolism and steroid biosynthesis, increased IL-6-mediated angiogenesis and tumor growth, stimulation of CCL2/IL-8 to promote tumor growth and cancer cell migration, and upregulation of IL11/FGF9 to activate STAT3, PI3K/AKT and MAPK signaling. AKR1C3 = aldo-ket reductase family 1 member C3; AKT = protein kinase B; AR = androgen receptor; ARSi = androgen receptor signaling inhibition; CCL2 = C_C motif ligand 2; CCR2 = C-C motif chemokine receptor 2; CXCL13 = C-X-C motif ligand 13; CXCR1/2 = C-X-C motif chemokine receptor 1/2; FGF-9 = fibroblast growth factor 9; DHT = dihydrotestosterone; FGFR = fibroblast growth factor receptor; FZD1 = Frizzled receptor 1; GAPs = GTPase activating proteins; GEFs = guanine nucleotide exchange factors; HMGCS2 = 3-hydroxy-3-methylglutaryl coenzyme A synthase 2; iGluR = inotropic glutamate receptor; IL-6 = interleukin 6; IL-8 = interleukin 8; IL-11 = interleukin 11; IL11RA = interleukin 11 receptor A; LMO2 = LIM domain only 2; MAPK = mitogen-activated protein kinase; PCa = prostate cancer; PI3K = phosphatidylinositol 3 kinase; RASAL3 = RAS protein activator like 3; SRFP1 = secreted frizzled-related protein 1; STAT3 = signal transducer and activator of transcription 3; TGFβ = transforming growth factor β; TNC = tenascin C; VEGF = vascular endothelial growth factor; WNT = wingless-related integration site. Created with Biorender.com (accessed on 1 December 2022) and data sourced from [5,6,7,191,228,257,258,259,260].

Hormone therapy using androgen receptor-signaling inhibitors (ARSi) is a standard treatment for patients with prostate cancer that have either relapsed after surgery and/or radiotherapy or present with metastatic disease at diagnosis [3]. However, despite initial responses to hormone therapy, resistance inevitably arises as the cancer evolves and transitions into CRPC (reviewed by [252]). While many mechanisms of resistance to ARSi have been discovered (e.g., *AR* amplification, *AR* mutations that increase AR transactivation, *AR* splice variants that lack a ligand-binding domain and AR bypass signaling, reviewed in [253,254,255,256]), recent evidence has begun to highlight the importance of fibroblasts and CAFs. Many mechanisms of CAF-induced resistance to androgen/AR-directed blockade have been explored in prostate cancer (Figure 1).

Numerous cells within the TME express AR, including CAFs, fibroblasts, endothelial cells, immune cells and disseminated prostate cancer cells [261], and AR signaling between the stromal and epithelial compartments is integral to normal prostate development and for the maintenance of normal adult prostate tissue homeostasis (as detailed in Section 1.1.1), tumorigenesis and CRPC growth [13,14,15,19,20,21,262]. Interestingly, orthotopic co-inoculation of AR-deficient human prostate stromal cells (WPMY1) and AR-negative CRPC cells (PC-3) in immunocompromised nude mice marginally reduced tumor burden relative to PC-3 tumors grown with human stromal cells expressing functional AR [262]. However, it is possible the marginal response observed reflects the presence of AR-positive host fibroblasts in the prostate. Moreover, co-deletion of AR in the prostate epithelium and systemically within the stroma of the TRAMP prostate cancer mouse model reduced metastatic burden in the lymph node compared to control TRAMP mice, whereas conditional AR loss specifically within TRAMP mouse prostate epithelial cells alone increased metastasis and reduced survival [262]. Conversely, in vivo tissue recombination models with primary prostate cancer cells combined with either AR-positive or AR-negative prostate myofibroblasts have shown that low stromal AR decreases castration-induced apoptosis and may impair invasion by maintaining an ECM microenvironment that suppresses cell movement [263]. Analysis of AR expression by IHC has also revealed that AR expression is reduced in prostate cancer-associated stroma relative to matched benign stroma, and negatively correlates with a high Gleason score [263], suggesting that stromal AR plays a tumor suppressive role. Taken together, these results highlight a complex relationship between fibroblast AR expression and prostate cancer growth, and emphasize the need for further work exploring the functional importance of stromal AR during prostate cancer and its predictive value.

A recent study has also shown that the LIM domain only 2 (*LMO2*, a key regulator of hematopoietic stem cell development [264]) is transcriptionally repressed by AR in prostate fibroblasts and that LMO2 is elevated in primary human prostate cancer vimentin^+^/αSMA^+^ CAFs in response to enzalutamide treatment [257]. LMO2 upregulation was found to augment CAF secretion of growth-promoting factors (e.g., IL-11 and FGF-9) to facilitate CRPC growth, reflecting non-cell-autonomous AR activation and elevated STAT3, AKT and ERK1/2 signaling [257]. Interestingly, stromal and epithelial cells also respond differently to testosterone-induced AR activation. Primary human vimentin^+^/α-SMA^+^/PDGFRβ^+^/AR^+^ CAFs have been shown to suppress the secretion of inflammatory/tumor-promoting cytokines (including CCL2 and CXCL8) in response to testosterone, whereas AR-positive LNCaP prostate cancer cells displayed AR nuclear translocation and activation of AR-mediated gene transcription [258]. Conversely, blocking AR signaling in CAFs enabled pro-migratory cytokine release, supporting prostate cancer cell growth and migration [258]. In combination, these data generally support the concept that the presence of AR in CAFs exerts a tumor suppressive effect during early, hormone-naïve stages of prostate cancer. In contrast, reduced expression of AR in CAFs, often in response to androgen/AR-pathway directed therapies, appears to promote tumor growth and progression, correlating with a high Gleason score, disease recurrence, and shorter progression-free survival [258,265,266,267].

Several recent studies have begun to explore how CAFs contribute to CRPC transition and have identified several therapeutic targets that could improve the efficacy of androgen/AR-pathway inhibitors. For instance, co-culturing human prostate cancer cells (DuCaP, LNCaP, LAPC4) as 3D spheroids with CAFs has been shown to reduce enzalutamide and bicalutamide sensitivity [5], and correlates with increased cholesterol metabolism and steroid biosynthesis in prostate cancer cells via the release of CAF-derived 3-hydroxy-3-methylgluteryl-Coenzyme A synthase 2 (HMGCS2) and aldo-keto reductase family 1 member C3 (AKR1C3) [260]. Notably, dual-targeting with simvastatin, a commonly prescribed inhibitor of cholesterol synthesis and an AKR1C3 inhibitor that blocks steroid biosynthesis were found to overcome enzalutamide resistance in this setting [260]. This indicates that targeting CAF-mediated metabolic regulation of prostate cancer cells may help overcome ARSi resistance. Accordingly, targeting androgen/AR signaling specifically within epithelial cells and not the stromal compartment, or therapies that enrich AR within the stroma are currently being investigated (reviewed in [268]). For example, the efficacy of agents targeting AR co-regulators and pioneer factors (transcription factors that can directly bind and open condensed chromatin to activate gene expression) that are active in epithelial cells and not fibroblasts/CAFs (e.g., FOXA1) is currently being investigated for the treatment of advanced prostate cancer [268,269,270].

In addition, CAF-derived IL-6 may also play a role during ARSi resistance. Given that IL-6 is exclusively produced in CAFs and not resting fibroblasts, it has been suggested that CAF-derived IL-6 may stimulate VEGF secretion from prostate cancer cells independently of AR signaling via PI3K/AKT, STAT3 and MAPK signaling [191,228]. Moreover, analysis of established prostate cancer cell lines (LNCaP, LAPC4, DuCaP) co-cultured with CAFs in 3D culture assays revealed CAFs caused upregulation of PI3K/AKT signaling within prostate cancer cells in response to androgen depletion [5]. Furthermore, treatment of these cells with a PI3K inhibitor (LY294002) was able to overcome ADT resistance [5]. It is also speculated that CAFs can facilitate prostate cancer metastatic potential in response to castration through the induction of EGFR/ERK signaling in prostate cancer cells to drive increased EMT and continued invasion and migration [271]. CAFs grown in a low-dihydrotestosterone (DHT), high-ethanol in vitro environment that mimics the prostate TME post-androgen ablation were shown to release EVs that harbor significantly reduced levels of miR-146a-5p than CAFs grown in the presence of androgens [271]. The role of miR-146a-5p in cancer is complex and cancer specific, however in prostate cancer it has been reported to induce a tumor suppressor function by stimulating downregulation of NF-kB and EGF signaling (reviewed in [272]). Consequently, diminishing miR-146a-5p levels in CAF-secreted EVs significantly increased prostate cancer migration, possibly reflecting EMT driven by the EGFR/ERK pathway [271].

Recently, CAFs have also been shown to promote CRPC growth by facilitating the emergence of treatment-induced neuroendocrine prostate cancer (t-NEPC), which occurs in approximately 10–15% of patients with advanced prostate cancer in response to ARSi [11,273]. Studies using 3D co-cultures (human CRPC 22Rv1 or PC-3 cells cultured with mouse primary wildtype prostatic fibroblasts) or co-engrafted xenografts (22Rv1 cells co-injected with CAFs) show that CD105 signaling in CAFs drives increased secretion of the Wnt regulator SFRP1 upon enzalutamide treatment, which correlates with expansion of the CD105-positive CAF population that promote neuroendocrine differentiation of adjacent prostate cancer epithelial cells in a paracrine manner [6]. Moreover, high numbers of CD105-positive CAFs in a range of prostate cancer specimens are reported to correlate with neuroendocrine differentiation in the clinic [6]. Nevertheless, further work is needed to fully comprehend the molecular mechanisms mediated by CD105-positive CAFs to drive t-NEPC and to determine if CD105-positive CAFs are indispensable for t-NEPC growth, offering a novel therapeutic avenue. Promisingly, co-treatment with enzalutamide and TRC105 (a partially humanized CD105 neutralizing antibody) is reported to suppress SFRP1 expression in 22Rv1 prostate cancer cells, circumventing neuroendocrine differentiation and suppressing CRPC progression in a xenograft mouse model that did not respond to either monotherapy [6].

Therapeutic resistance to ARSi may also occur through epigenetic changes in CAFs that instigate a cascade of altered stromal-epithelial interactions. For example, epigenetic silencing of the Ras inhibitor *RASAL3* in human prostate CAFs is reported to occur in response to enzalutamide and bicalutamide treatment both in vitro and in vivo [259]. *RASAL3* silencing in CAFs increases oncogenic Ras activity and upregulates glutamine synthesis. Ultimately, glutamine secretion by CAFs serves as an energy source for prostate cancer cells through anaplerosis causing metabolic reprogramming via elevated mTOR signaling in prostate cancer cells to facilitate CRPC growth and neuroendocrine differentiation of prostate adenocarcinoma [259].

In combination, these findings highlight the functional importance of CAFs during ARSi resistance, and illustrate that CAFs are also responsive to androgen levels. Hence, combining ARSi with agents that either target pro-tumorigenic CAF activities or permit CAF AR signaling may block or delay CRPC growth.

### 5.2. CAFs and Chemoresistance

**Figure 2:**
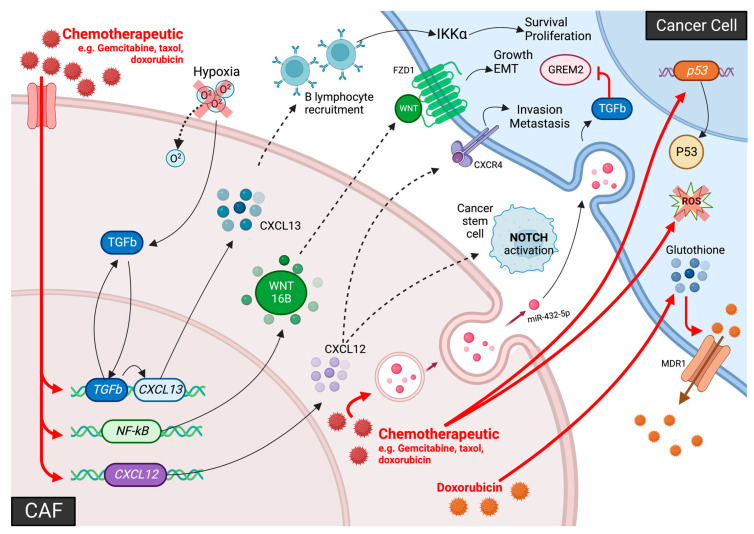
Overview of potential CAF-regulated resistance mechanisms to chemotherapy. Schematic shows the diverse molecular mechanisms that have been identified in the literature to be regulated by CAFs in response to chemotherapy in multiple cancers, including prostate cancer. These include hypoxia-induced TGFβ-CXCL13 signaling that increases B-cell recruitment, augmented NF-kB-WNT16B-β-catenin signaling in response to genotoxic stress, CXCL12-CXCR4, CXCL12-Notch oncogenic signaling, p53 attenuation and deregulation of glutathione metabolism and secretion of EVs carrying miR-432-5p to activate TGFβ signaling. CXCL12 = stromal-derived factor 1; CXCL13 = C-X-C motif ligand 13; EMT = epithelial to mesenchymal transition; FZD1 = Frizzled receptor 1; GREM2 = gremlin 2 DAN family BMP antagonist; IKKα = inhibitor or nuclear factor kapper B kinase complex; MDR1 = multidrug resistance 1 protein; NF-κB = nuclear factor kappa B; ROS = reactive oxygen species; TGFβ = transforming growth factor β; WNT = wingless-related integration site. Created with Biorender.com (accessed 1 December 2022), data sourced from [7,189,275,276,277,278,279,280,281,282,283,284,285,286,287].

Treatment regimens for patients that develop advanced CRPC, or patients that present with metastatic disease include chemotherapy (e.g., docetaxel or cabazitaxel) in combination with ARSi and/or targeted therapies being explored in a clinical trial (e.g., PARP or AKT inhibitors) [274]. Several studies have explored the role of CAFs during chemorefractory disease, establishing that CAFs can also facilitate chemotherapy resistance via a range of molecular mechanisms (summarized in Figure 2), including those that have been shown to promote CRPC growth. For example, CXCL13 expression in CAFs positively correlates with worse prostate cancer severity in prostate cancer patients and is elevated in response to ASRi and/or chemotherapy [275]. Moreover, prostate intratumoral hypoxia and induction of autocrine TGFβ signaling in response to ASRi or chemotherapy promotes CAF activity and their subsequent secretion of CXCL13, leading to the recruitment of B lymphocytes that promote therapeutic resistance via secretion of inflammatory cytokines, which activates inhibitor of nuclear factor kappa-B kinase subunit alpha (IKKα) within CRPC cells to promote survival and proliferation [275,276]. Furthermore, pharmacological intervention with a phosphodiesterase 5 (PDE5) inhibitor or a TGFβR inhibitor reduced myofibroblast activation and CXCL13 induction, which prevented CRPC growth in the TRAMP model [275]. Thus, targeting CAF-mediated CXCL13 secretion may provide a unique treatment approach to prevent/delay CRPC and chemorefractory disease.

Several studies encompassing multiple cancer types, including prostate, have also reported that signaling molecules secreted by CAFs can promote cancer cell survival and proliferation when exposed to chemotherapeutic agents [277,288] (summarized in Figure 2). For instance, the upregulation of CAF-derived WNT16B secretion via NF-kB signaling has been linked to genotoxic stress, the subsequent activation of canonical Wnt/β-catenin pathway to promote chemo-refractory tumor growth and EMT [277]. Moreover, siRNA-mediated silencing of WNT16B, β-catenin or NF-kB signaling in CAFs engrafted with PC-3 cells in xenografts prevented chemotherapy resistance [277], highlighting the potential therapeutic benefit of targeting the CAF secretome in combination with chemotherapy. In addition, studies in breast cancer patient-derived xenografts (PDX) have also shown that IL-6 and IL-8 release by CD10^+^ GPR77^+^ CAFs increases cancer stem cell activity via persistent activation of NF-κB to support tumor survival and chemotherapy resistance [163].

In prostate cancer, CAFs are also reported to facilitate chemotherapeutic resistance via attenuation of p53. Chetah and colleagues found that co-culturing of LNCaP prostate cancer cells with primary human CAFs or CAF CM reduced p53 in prostate cancer cells, which inhibited drug-induced ROS in response to doxorubicin, taxol or mitomycin C to promote cell survival [278]. Furthermore, following doxorubicin treatment LNCaP cells exhibited a significant decrease in DNA damage and decreased drug accumulation [278]. CAF-driven chemotherapy resistance in this model also caused an elevation in LNCaP glutathione levels [278], suggesting that glutathione may facilitate doxorubicin export, possibly via stimulation of multidrug resistance 1 protein (MDR1) [280,281,286]. CAF-secreted EVs may also play a role in facilitating chemotherapy resistance, as EVs isolated from primary human vimentin^+^/αSMA^+^/FAP^+^ CAFs in cell culture containing miR-432-5p have been shown to inhibit gremlin 2 DAN family BMP antagonist (GREM2) and promote TGFβ signaling in prostate cancer cells to drive chemoresistance [283].

During prostate cancer metastasis, a CXCL12-rich niche is reported to facilitate colonization at a secondary site (particularly in the bone) and CXCR4-CXCL12 signaling plays a key role in mediating tumor–stroma interactions required for prostate cancer cells to grow in the presence of chemotherapeutic agents [189,284,287]. Bone-derived CAFs express CXCL12 which directly stimulates the CXCR4 receptor on the surface of prostate cancer cells to elicit a protective effect from chemotherapy, as well as promoting metastatic progression and attracting inflammatory immune cells to the TME [284,289]. The mechanism by which CXCR4 stimulates its chemoprotective effect is currently unknown, however CXCR4 is thought to facilitate multiple stages of the metastatic cascade, including homing to the metastatic niche and colonization [279]. Furthermore, co-treatment of AMD3100 (a CXCR4 inhibitor) and docetaxel significantly reduces tumor growth in the PC-3 CRPC xenograft mouse model relative to monotherapy and can sensitize PC-3 cells to docetaxel [289]. Intriguingly, CXCL12 has also been shown to operate in an autocrine positive feedback loop to maintain an oncogenic CAF phenotype in breast cancer, where CAF-derived CXCL12 was also found to regulate cancer stem cells through activation of the NOTCH pathway [282,285]. Together, these findings highlight an important role for CAF-derived CXCL12 during tumor progression and chemoresistance, and indicate that inhibition of the CXCL12/CXCR4 signaling axis may chemo-sensitize prostate cancer cells. However, to date, CXCR4 inhibitors remain to be explored in the clinic. Nevertheless, drugs that reprogram CAFs are beginning to enter clinical trials and are starting to show promise in combination with gemcitabine in pancreatic cancer [14].

### 5.3. Immune Checkpoint Inhibitor Resistance

Prostate tumors are generally regarded as immunologically “cold”, which means they respond poorly to immunotherapy owing to low T cell infiltration [244,248]. Immunotherapies, such as ICB that acts to inhibit cytotoxic T-lymphocyte antigen 4 (CTLA-4), programed cell death protein 1 (PD-1) and its ligand (PD-L1) have shown efficacy against “hot” tumors, and accordingly significant research effort has been spent on identifying ways to switch the TME of a “cold” tumor to “hot” [290]. Remarkably, CAFs can also remodel the ECM to create a physical barrier to infiltrating immune cells [291,292], and analysis of TCGA RNA-seq data from 15 different tumor types has revealed that deregulation of ECM-related genes in CAFs correlates with increased TGFβ signaling and insensitivity to ICB [251], suggesting that CAF coordinated creation of a reactive stromal TME, via ECM remodeling and TGFβ signaling, contributes to the development of a cold immune microenvironment in the prostate, thus rendering ICB ineffective.

A recent study has also indicated TGFβ^+^/TGFβR2^+^ CAFs may facilitate ICB resistance. Human metastatic urothelial tumor samples (*n* = 298) that had been treated with an anti-PD-L1 antibody (atezolizumab) showed a positive correlation between reduced treatment response and high TGFβ signaling in CAFs [293]. Indeed, histological and gene expression analysis revealed increased CAF-mediated ECM deposition in the TME resulted in the production of a ‘stromal barrier’ that causes physical exclusion of CD8^+^ T cells, a characteristic of an immune excluded microenvironment [293]. Furthermore, therapeutic blockade of PD-L1 and TGFβ in an immune-excluded mammary mouse model (EMT6) led to a significant increase in infiltrating T cells as well as tumor regression and suppression of metastasis [293]. Similarly, treatment with ICB has been shown to suppress metastasis in a colorectal cancer transgenic mouse model with TGFβR2 depletion (*Lgr5^eGFP-creERT2^*, *Apc^fl/fl^*, *Kras^LSL-G12D^*, *Tgfbr2^fl/f^*, *Trp53^fl/fl^)* [294].

CAF-derived CXCL12 has also recently been implicated in immunotherapy resistance. Using a KPC mouse model of PDAC that is insensitive to ICB, it has been revealed that depletion of FAP^+^ CAFs (by targeting human diphtheria toxin receptor where expression is driven by a modified *FAP* gene) can increase sensitivity to anti-CTLA-4 and anti-PD-L1 therapy, via a CXCL12-dependent mechanism [295]. Indeed, co-treatment of a CXCR4 inhibitor (AMD3100) with ICB in PDAC mice with an intact FAP^+^ CAFs population induced rapid T cell accumulation and significant tumor regression [295]. Many subpopulations of prostate CAFs express CXCL12 [26,57,77,154,247], potentially protecting prostate cancer cells from T cell mediated destruction and causing a lack of response to ICB, thus targeting CXCL12-expressing CAFs offers and exciting new treatment avenue to circumvent immune evasion and increase ICB sensitivity.

## 6. Targeting and Reprogramming of CAFs

Drug resistance to several other targeted therapies being explored in the clinic has also recently been linked to CAF activity. For example, BRAF inhibition in melanoma patients has been shown to cause CAF-mediated ECM remodeling, which supports melanoma growth and promotes BRAF-inhibitor resistance by upregulating integrin β1/FAK/Src signaling [296]. Additionally, αSMA^+^/PDGFRβ^+^ CAFs have also been shown to desensitize prostate cancer cells to the multi-tyrosine kinase inhibitor sorafenib by mediating ERK phosphorylation/activity in prostate cancer cell lines (22Rv1, PC-3), increasing their ability to evade sorafenib-induced apoptosis and permit drug-resistant growth [297]. Given the role of CAFs in facilitating drug-resistance, several research groups have begun to unravel the complex regulatory pathways underpinning fibroblast activation to identify novel therapeutic approaches to block CAF generation, recruitment and activation. Recent findings for instance have shown that high protein expression of yes-associated protein 1 (YAP1) in prostate fibroblasts can induce their differentiation into FAP^+^/αSMA^+^ CAFs [298]. YAP1-mediated fibroblast-to-CAF transition is reported to involve the upregulation of *SRC* transcription due to the formation of a YAP1/TEAD1 protein complex which binds to and stimulates the promoter region of *SRC*, which in turn elevates the transcription of *SRC* and SRC target genes that regulate cytoskeletal proteins and actins to maintain a CAF phenotype [123,298]. High protein expression of YAP1 and SRC are commonly observed in high Gleason grade prostate cancer clinical specimens, and positively correlates with a poor prognosis and metastatic burden [298,299], indicating that YAP1 and SRC may provide valuable therapeutic targets and warrant further investigation. Importantly, reprogramming CAFs using a CAF-targeting siRNA delivery system is reported to show efficacy preclinically. For instance, a CAF-targeting nanoparticle siRNA delivery system whereby FAPα antibody is loaded onto cell-penetrating peptide (CPP)-based nanoparticles to deliver siRNAs targeting the CAF-derived chemokine *CXCL12* is reported to significantly inhibit tumor cell migration, invasion and angiogenesis in xenografts where PC-3 prostate cancer cells and FAP^+^ CAFs were co-engrafted orthotopically into nude mice [284,287,289,300].

Inhibition of FGF2, a mitogenic factor for fibroblasts, is also currently being explored as a novel treatment avenue to block CAF function, and has shown promise in multiple cancers preclinically [301,302,303]. In breast cancer, *FGF2* mRNA expression positively correlates with CAF abundance, and siRNA silencing of *FGFR1* in MDA-MB-231 breast cancer cells co-implanted with FGF2^+^ CAFs significantly reduced xenograft tumor burden [304]. FGF2 is also upregulated in prostate cancer and a ribozyme-targeting approach to selectively deplete FGF-binding protein (FGF-BP) to reduce FGF2 mobilization and activation from the ECM has been shown to prevent PC-3 xenograft tumor formation in athymic nude mice [301]. Targeting FGFR1 with a small molecule allosteric inhibitor (SSR128129E) is also being investigated, however only limited efficacy has been observed to date [302].

The fibroblast marker FAP is a serine protease commonly expressed on the surface of reactive stromal CAFs that is generally considered to promote tumorigenesis (possibly more so than mature, fully differentiated CAFs that express FAP infrequently) [25,74,305,306]. FAP-expressing CAFs have been detected in multiple epithelial tumors, including prostate cancer, and have also been explored as a therapeutic target [25,74,305,306,307]. A phase I clinical trial has confirmed the safe uptake of an unconjugated humanized FAP monoclonal blocking antibody (sibrotuzumab) to target FAP^+^ fibroblasts in colorectal carcinoma and non-small cell lung cancer, although there was very little tumor response and the antibody has not progressed in the clinic [308]. Interestingly, FAP gelatinase and collagenase endopeptidase activity has also been exploited to activate prodrugs and protoxins. Intratumoral injections of a newly synthesized FAP-activated protoxin caused cell lysis of FAP^+^ CAFs and reduced tumor growth in breast and prostate cancer xenografts [307]. Furthermore, a FAP-activated prodrug derived from thapsigargin (a highly toxic natural plant product that stimulates increased intracellular calcium levels to cause apoptosis) is also reported to decrease tumor burden in LNCaP prostate cancer xenografts [309].

Given the complexity of CAF signaling and the fact that CAFs can mediate both oncogenic and tumor suppressive functions depending on the tumor subtype/stage, therapeutic interventions that can reprogram CAFs are also being explored in several solid cancers, that may also prove to be efficacious in prostate cancer. For instance, targeting CAFs using an endo180 targeting antibody drug conjugate (ADC) is reported to be efficacious against sarcoma, glioblastoma and leukemia without toxicity in preclinical trials in vivo [310]. It will be important for future work to determine if prostate endo180^+^ CAFs exist and if so, to explore if endo-180 ADC therapy is also effective in prostate cancer. Furthermore, vitamin D receptor (VDR) directed therapy has been shown to reprogram CAFs into a quiescent state and treatment using the VDR ligand analogue calcipotriol is reported to improve the delivery and efficacy of gemcitabine, where combination therapy significantly reduced tumor burden relative to monotherapy in both gemcitabine responsive and non-responsive PDAC mouse models [311]. The BET inhibitor JQ1 has also been shown to reduce Hh and TGFβ signaling in PDAC CAFs to suppress CAF activity and can suppress tumor growth and induce stromal remodeling in PDAC PDXs [312].

In summary, CAFs present an attractive target to suppress tumor growth, progression and therapeutic resistance, and evidence to date indicates that CAF-directed therapies may benefit patients. However, a deeper understanding of CAF activation, distribution and functions is needed to enable optimal targeting of CAFs. Advantageously, αSMA^+^/PDGFRα^+^ CAFs appear to preferentially position themselves around young blood vessels making them an ideal target for drug delivery [313,314]. Consequently, stromal cells could be exposed to therapeutic agents delivered via the circulation before the tumor cells [315]. The impact of stromal remodeling and ECM stiffness on drug penetration and accumulation within the TME will also need to be taken into consideration as a stiffer ECM could hinder drug delivery [11,188]. In addition, while diminishing FAP^+^ CAFs in mice has been shown to reduce tumor burden in melanoma and pancreatic cancer models, associated with impaired immunosuppressive functions and reduced angiogenesis, respectively [295,316,317], it has also been shown to cause adverse side effects such as cachexia and anemia in a transgenic mouse model [318]. It is therefore possible that these adverse effects may also arise in future clinic trials exploring CAF-targeted therapies, particularly if the treatment is delivered systemically. In corroboration, these findings emphasize that CAF-directed therapies have therapeutic potential and may sensitize CAFs to ARSi, chemotherapy and/or immunotherapy, yet more work is needed to determine how they can be optimally targeted with a clinically manageable safety profile.

## 7. Conclusions

Primary, localized prostate cancer is most often successfully cured with treatments, such as surgery, hormone therapy and/or radiotherapy. However, a small subset of patients will acquire resistance to these therapies and manifest metastatic CRPC, a lethal disease with a median survival of less than 5 years [1,2,3,4,5,6]; therein, highlighting the current lack of prognostic tools to identify patients at risk of developing castration-resistant metastatic disease and emphasizing the need for new preventative/curative treatments. While the majority of prostate cancer research has focused on understanding epithelial cell biology, in this review we highlight the diverse nature of CAF subtypes within the TME of the prostate, and their multifunctional importance during prostate cancer formation, progression and drug-resistance. This leads us to recognize that CAFs are actionable therapeutic targets with considerable future potential to restrain and/or diminish tumor growth, thus providing a new avenue of treatment to improve patient health.

Currently however, the complexity of the mesenchymal cell network within the TME, and the unavailability of clear-cut molecular markers that well-define particular CAF-subsets, present considerable challenges in terms of our capacity to optimally manipulate the stroma and effectively target the TME. Defining the diversity of CAF origins, arising from local or infiltrating cells, and understanding their relative pro- or anti-tumorigenic contributions also requires further investigation. To this end, it will be important for future work to determine if promoting fibroblast specific phenotypes that exert acute inflammatory responses and limit ECM stiffening, whilst encapsulating the tumor cells is beneficial for patient health. Conversely, suppressing the activities of CAFs that drive pro-fibrotic like effects, limit immune-cell functionality and chronically persist to modify the tissue architecture would also be an attractive goal. However, we must note the vital importance of the fibroblasts as sentinel orchestrators of homeostasis in organ systems throughout the body. Hence, systemic therapeutic interventions to manipulate fibroblasts may cause adverse effects that limit the efficacy of fibroblast-targeted therapies against prostate cancer and other malignancies.

Nonetheless, in the study of CAF-phenotypes, a better understanding of the diversity and nuances of the stromal-cell populations and their specific functional roles in tumor tissue remains important. Considerable additional information can be exploited by exploring not only the profiles of the epithelial compartment, but adding to this detailed descriptors of the CAF-population provides a fuller picture of aberrations of the microenvironment. Technologies centered on spatial interrogation of tissues, in terms of cell clusters or at a single cell level are increasingly being employed, revealing the importance of molecular phenotypes and their spatial distributions in tissues. This additional intel holds promise for more accurate predictions of aggressive prostate cancer, aiding treatment decisions for high-risk localized disease, whilst alleviating the burden of over-treatment of men with slow-growing cancers. Furthermore, defining relationships between CAF-subsets and aberrantly activated oncogenic signaling pathways will be essential for the rational design of interventions which target both epithelial and stromal compartments.

Ultimately, delineating the true nature of the CAF populations, to understand if these are potent in promoting tumor growth and progression, is paramount for future advances in molecular pathology, diagnosis and risk prediction, and the application of stromal-modulating agents. This field continues to evolve at pace, and offers exciting opportunities to develop an arsenal of therapeutic options suitable for prostate and various other solid cancer to improve clinical outcomes.

## Data Availability

Not applicable.
